# Proton sponge: an aromatic glycolysis catalyst

**DOI:** 10.1039/d6ra00723f

**Published:** 2026-02-09

**Authors:** Robbie A. Clark, Ciaran W. Lahive, Michael P. Shaver

**Affiliations:** a Sustainable Materials Innovation Hub, Henry Royce Institute, University of Manchester Manchester M13 9BL UK michael.shaver@manchester.ac.uk

## Abstract

Chemical depolymerisation of poly(ethylene terephthalate)(PET) is a widely explored method to recycle plastic waste, with particular benefits on waste streams unsuitable for mechanical recycling. Glycolysis, the employment of ethylene glycol (EG) and a catalyst to effect depolymerisation, is a promising technology. Herein, we report the use of 1,8-bis(dimethylamino)naphthalene, commonly known as a proton sponge (PS), as an effective, novel organocatalyst for PET glycolysis. Use of PS enables an 89% bis(2-hydroxyethyl)terephthalate (BHET) yield after only 45 min at 180 °C using 10 equiv. EG and 20 mol% catalyst. The aromaticity of PS allows for a shortened induction time by improving PET swelling compared to comparably basic non-aromatic catalysts such as tributylamine and pempidine. PS glycolysis obeyed pseudo first-order kinetics (*R*^2^ > 0.98) with an apparent activation energy of 126.3 kJ mol^−1^. Depolymerisation catalysed by PS is shown to be tolerant of air and a reduced catalyst loading of 5 mol%, and was demonstrated at 10 g scale, giving a 64% BHET isolated yield (>99% purity). A range of aromatic amines, structurally related to PS, were synthesised and investigated to provide a deeper understanding and mechanistic insights into the reactivity of this class of amine catalyst.

## Abstract 2

Glycolysis of poly(ethylene terephthalate) by aromatic catalysts has the potential to expedite reactions times by enhancing catalyst–substrate interactions.

## Graphical abstract

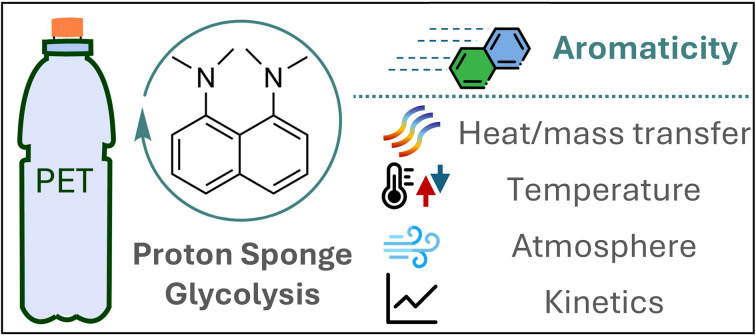

## Introduction

Plastics are diverse in both applications and properties, with tunable tensile strength, density, stability, barrier properties, printability, form and impact resistance contributing to their ubiquity.^1^ However, the mismanagement of plastics is exacerbating growing environmental and societal injustice. Resultant greenhouse gas release, public health impacts of littered and burned plastics, harm to wildlife, pollution of natural habitats, and a growing trend of micro and nano-plastics being found within human bodies,^2–7^ are alarming. A range of recycling technologies are needed to address these plastics challenges; while mechanical recycling is important for clean mono-materials, chemical depolymerisation can furnish highly pure compounds from compositionally varied plastic streams.^8,9^ These products are amenable to repolymerisation into virgin-quality polymers, closing the loop on consumption of finite resources. Within this space, a range of organocatalysts have been explored.^9–11^

Organocatalysts have been prominently investigated for the depolymerisation of poly(ethylene terephthalate) (PET),^12^ a polyester which makes up a large proportion of plastic waste.^13,14^ Many catalysts promote PET breakdown through basicity, engaging in hydrogen bonding with alcohols like ethylene glycol (EG), weakening its O–H bonds to promote attack.^15,16^ Catalysts with rising basicity, like dimethylaniline (DMA), *N*-methylimidazole (NMI), 4-dimethylaminopyridine (DMAP) and 1,8-diazabicyclo[5.4.0]undec-7-ene (DBU) (p*K*_a_ (H_2_O) = 5.07, 7.4, 9.7, 11.9 respectively) promote concomitantly rising reaction rates.^17^ Some catalysts even function through a dual hydrogen-bonding mechanism, with EG molecules coordinating carbonyl activation.^15^ Efforts to improve PET glycolysis have also sought to overcome its limited solubility and swelling in ethylene glycol.^11,18^ Addition of aromatic cosolvents like anisole can accelerate PET breakdown.^19^ Cosolvents may, however, increase potential environmental and economic burdens by requiring additional separation and recovery processes.^9,20^ Herein, we sought to combine the basicity of catalysts like DBU with the beneficial aromaticity of cosolvents like anisole. While many aromatic alkyl amines are weaker bases due to the electron-withdrawing effect of attached aromatic moieties, we sought to overcome this by taking advantage of the “proton sponge” effect of 1,8-bis(dimethylamino)naphthalene (Proton Sponge, PS).^21^ Its unique structural features (*N*–*N* proximity, alignment and steric clash), PS is a much stronger base than it otherwise would be. During our exploration of PS's performance, we sought to rationalise its performance by isolating the effect of aromaticity and by comparison to a synthesised library of aromatic alkyl amines.

## Experimental

### Materials

Colourless, clear pre-consumer PET bottles were manually cut into squares (dimensions = 3.74 ± 1.32 mm *×* 3.24 ± 0.90 mm, thickness = 0.30 ± 0.04 mm, surface area = 27.96 ± 10.67 mm^2^, Table S1), rinsed three times with deionised water and dried for at least 16 h in a 120 °C Fistreem OVA031.XX.3.5 vacuum oven. Colourless clear pre-consumer PET pots, tubs and trays were shredded before depolymerisation (using a SDS Shredder Basic Machine). The following chemicals were used as received from Sigma-Aldrich (methyl iodide, ethyl acetate, 1,8-bis(dimethyl-amino)naphthalene, trifluoroacetic acid-d_1_, pempidine, anhydrous ethylene glycol, potassium hydroxide, dimethyl sulfoxide, 1,2-phenylenediamine, 2,3-diaminotoluene), Thermo-Fisher Scientific (dimethyl sulfoxide-d_6_, tributylamine, diphenyl ether, 1,3-phenylenediamine and 1,4-phenylenediamine) Alfa Aesar (8-aminoquinoline and 1,5-diaminonaphthalene; VWR (anhydrous dimethylformamide) and Honeywell (chloroform).

## Methods

All reagent equivalents (equiv. or mol%) are stated relative to PET repeat units (*i.e.* PET mass divided by 192.17 g mol^−1^). Unless otherwise stated, all reactions were conducted at 0.3 g PET scale. The bottle-grade PET used herein was characterised by size, thermal transitions, molar mass, crystallinity and thermal stability (Fig. S1–S4). All reactions, other than the scaled-up trials, were carried out in sealed microwave vials rated to withstand up to 30 bar. All yields are totals of BHET and BHET dimer. For details of characterisation methods and instrumentation see SI.

### Glycolysis of poly(ethylene terephthalate)

Poly(ethylene terephthalate) (0.3 g), ethylene glycol (0.969 g, 10 equiv.) and PS (0.0669 g) were weighed into a 20 ml microwave vial along with a stirrer bar. The vial was sealed, and nitrogen was flushed through the headspace for 1 min. The microwave vial was placed in a pre-heated oil bath at 180 °C and stirred at 150 rpm for the desired duration. The vial was then removed from the oil bath and allowed to cool to room temperature (∼25 °C). The vial was unsealed, 2 ml of DMSO added, and then stirred for 45 min until all solids dissolved excluding residual PET. At this point, a 100 µL sample of the mixture was transferred into an NMR tube along with 400 µL of a DMSO-*d*_6_ stock solution containing a known amount of an internal standard, diphenyl ether (DPE), and analysed. For details of the scaled-up method, see SI.

### Methylation of aromatic amines

This procedure was adapted from Sorokin *et al.*^22^ An amine substrate (7 mmol), KOH (28 or 42 mmol, equalling MeI moles), and DMF (7.5 ml) were weighed into a 20 ml microwave vial. A stirrer bar was added and the vial sealed. The vial headspace was flushed with N_2_ for 1 min after which MeI (28 or 42 mmol) was slowly syringed into the sealed vial. The vial was then placed into a preheated oil bath at 100 °C and stirred for 16 h at 150 rpm. The vial was cooled to room temperature, and the pressure released by puncture. Deionised water (10 ml) was added to dissolve KI and transfer contents into a separatory funnel. The vial was also rinsed with a small quantity of acetone (2 ml). A 20% m/v KOH solution (40 ml) was added to the separatory funnel. The basic aqueous phase was extracted with ethyl acetate (3 × 50 ml). The organic phase was then removed and dried over magnesium sulfate, before being reduced under rotary evaporation to yield the aromatic amine product. Full characterisation of aromatic amines 2–7 are provided in the SI.

### Quantitative NMR (Q-NMR)

To calculate the yield of BHET by Q-NMR (*i.e.*^1^H), the integral of a BHET peak (m, 4H, 8.03–8.17 ppm) was compared to the integral of a diphenyl ether internal standard peak (t, 2H, 7.13 ppm), along with the moles of diphenyl ether within the NMR sample. A similar method was used to quantify the BHET dimer. Further details are provided in the SI.

## Results and discussion

**Fig. 1:**
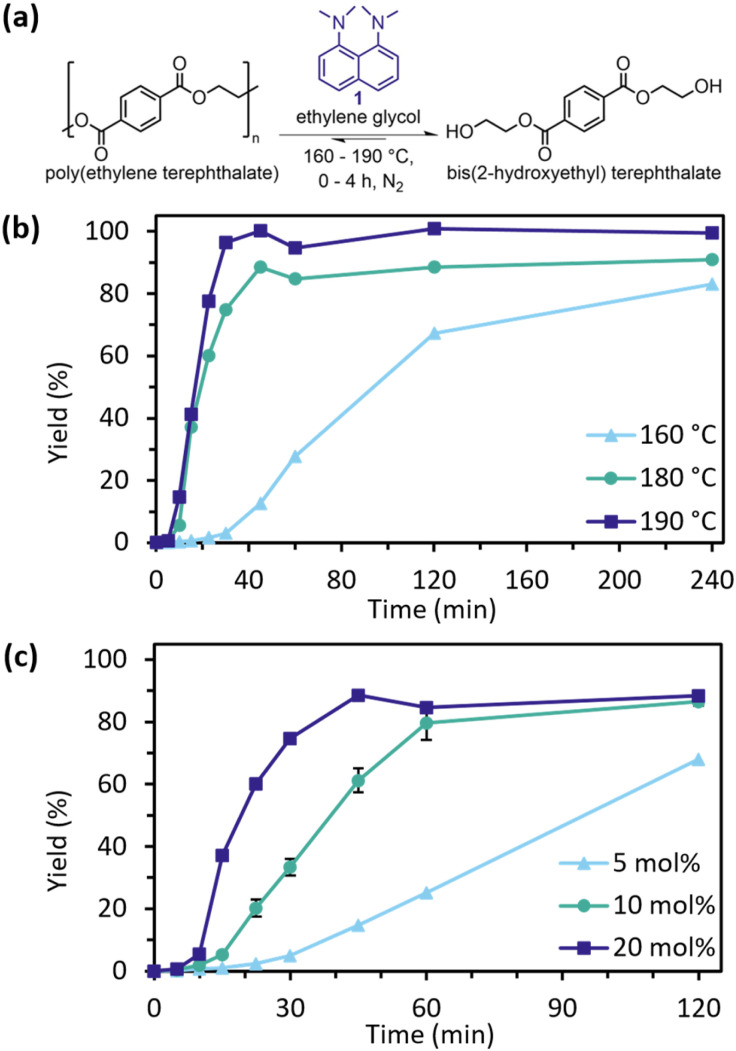
(a) PET glycolysis catalysed by PS. (b) Effect of temperature at 20 mol% catalyst, 10 equiv. EG. (c) Effect of catalyst loading (PS in mol%), at 10 equiv. EG and 180 °C. 10 mol% was triplicated with standard deviation shown. Yield (%) is the amount of PET converted to BHET or its dimer.

Screening for glycolytic activity of bottle-grade PET by PS used 20 mol% catalyst and 10 equiv. EG under N_2_ at 180 °C (Fig. 1a and b) giving 89% yield in 45 min, raising to 100% yields at 190 °C while decreasing to 83% at 160 °C even after extending to 4 h reaction times. This temperature-yield dependence has been observed elsewhere and reflects thermodynamic control of the position of equilibrium.^11^ The effect of catalyst concentration was also explored (Fig. 1c). Reducing from 20 mol% to 10 mol% loading was beneficial, as similar yields were attained at 60 min, although the reaction was slower.

**Fig. 2:**
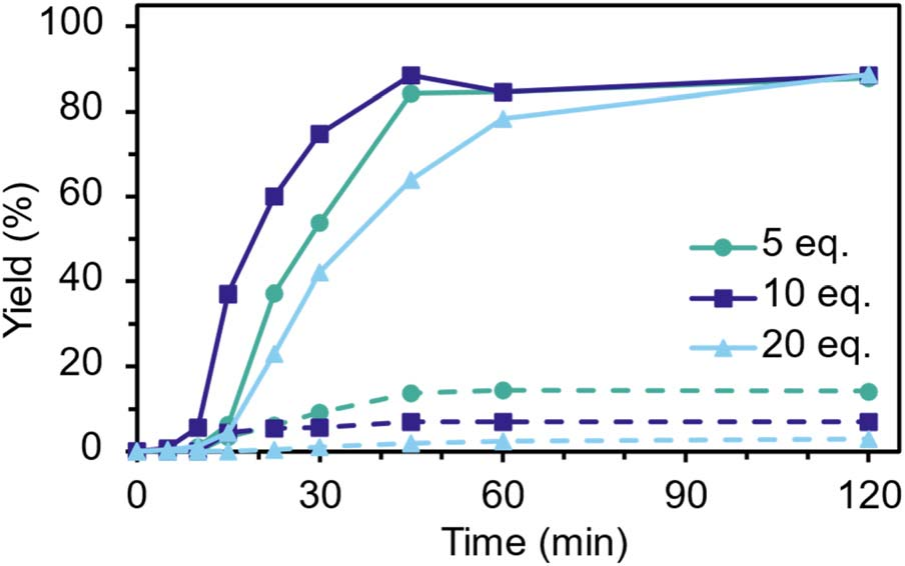
Effect of ethylene glycol loading. The total yield of BHET and its dimer is shown in solid, and the dashed line shows dimer proportion (at 20 mol% PS and 180 °C).

Reducing the catalyst loading further to 5 mol% reduced yields to 68% even after 120 min. Ethylene glycol ratios were investigated and revealed an interesting performance trade-off (Fig. 2). With 10 equiv. of EG, high yields at 45 min were produced, but contained 7% BHET dimer as a less desirable end-product. Increasing EG to 20 equiv. decreased dimer retention to ∼2%, but the resultant decreased catalyst concentration decreased rates. Decreasing the EG to 5 equiv. produced more oligomer (14%) and had an intermediate reaction rate. In this case, higher catalyst concentration is counteracted by insufficient EG availability for both reactivity and PET wetting. The EG-loading to dimer-production dependence is in line with previous reports.^23^ Fundamentally, the balance between the initial supply of EG pushing the PET polymer–dimer–monomer equilibrium towards monomer and altering catalyst concentration is key.

**Fig. 3:**
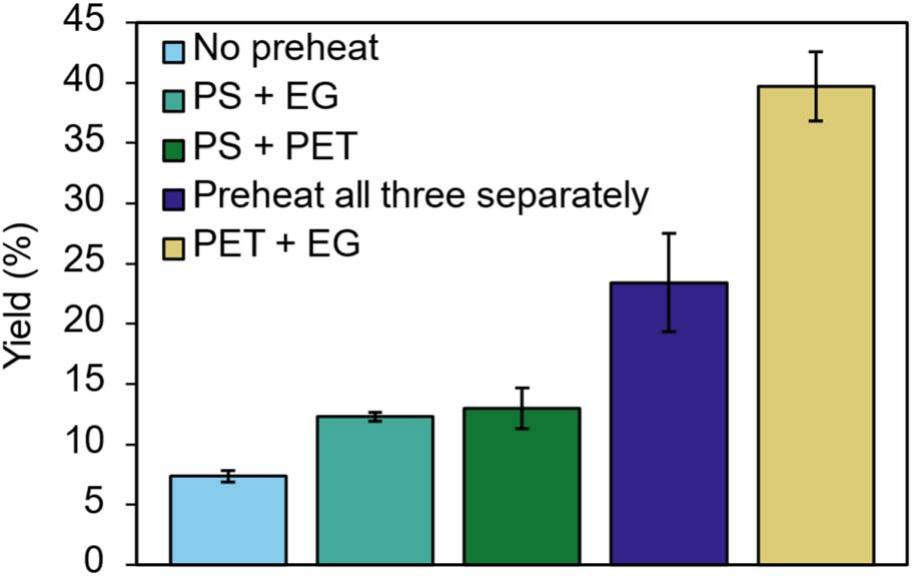
Preheating experiments conducted at 5 equiv. EG and 20 mol% catalyst, 180 °C, with a 15 min preheat followed by a 15 min reaction time. Each condition measured in triplicate, standard deviation shown. For more details see SI.

Of note, glycolysis reactions highlighted in Fig. 1 often contained induction periods. To understand this phenomenon, we developed a series of preheating experiments where combinations of catalyst, substrate and ethylene glycol were heated together before the addition of the third (Fig. 3) and compared to controls with fully separate or no preheating.

**Fig. 4:**
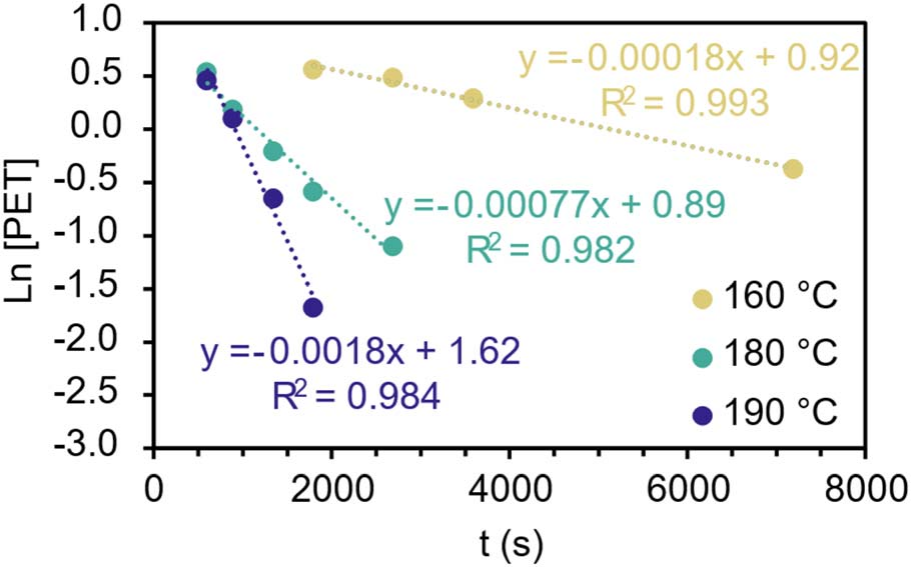
Kinetic plots showing first-order reaction dependence from runs at 0.3 g PET, 20 mol% PS, 10 equiv. EG and 3 temperatures (160 °C, 180 °C and 190 °C).

These experiments showed that across all cases, preheating increased the reaction yield at 15 minutes from 7%–40%, suggesting that heat-transfer limitations may cause this induction period. The combination of PET and EG proved even more successful (40% yield after 15 min) than preheating all three separately, suggesting an additional synergistic effect. We hypothesise that PET-swelling by EG entering the polymer matrix and creating space for catalyst molecules to flow is important. This finding corresponds with other reports of glycolysis enabled within a portion of PET that is swollen, not just at the surface.^11^ To compliment this understanding of the induction period, we used kinetic and Arrhenius plots to understand the kinetics of PS-catalysed glycolysis (Fig. 4 and S33). At 160 °C, 180 °C and 190 °C, a pseudo first-order (PFO) kinetic treatment modelled our results well (*i.e. R*^2^ > 0.98) giving a calculated activation energy for PS of 126.3 kJ mol^−1^. This contrasts with studies that found that shrinking-core kinetics provide a better fit.^11^ We would rationalise this behaviour by the high-surface area-to-volume ratio of our PET substrate, exemplified by the low average sample thickness (0.3 mm, Table S1).

As the PS glycolysis kinetics were well-modelled by a PFO treatment, this suggests mass-transfer limitations can be largely neglected and are therefore not significantly rate limiting after the induction period has ended. Combining this finding with our preheating experiments, we conclude that the induction period is compounded by both heat-transfer and swelling limitations. We would highlight that kinetic comparisons of depolymerisation catalysts is challenging, due to differing kinetic treatments, PET substrates and pre-treatments (outside of preheating the samples used are simply manually cut bottles).

**Fig. 5:**
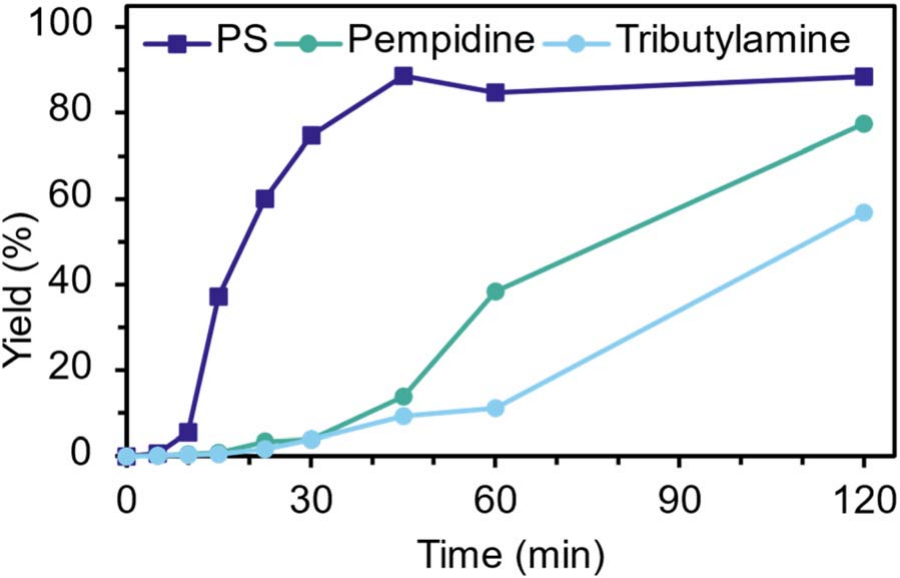
Comparison of PS and tributylamine at 20 mol%, 10 equiv. EG and 180 °C.

With baseline optimisation of PS glycolysis conditions complete, we sought to investigate the impact of PS aromaticity on the catalysis. To do so, PS was compared to two organocatalysts, pempidine and tributylamine (Fig. 5). Pempidine (p*K*_a_ = 18.2, ACN)^24^ and tributylamine (TBA) (p*K*_a_ = 18.1, ACN)^25^ were selected due to their similar basicity to PS (p*K*_a_ = 18.2–18.7, ACN)^26,27^ while containing tertiary alkyl amines.

**Fig. 6:**
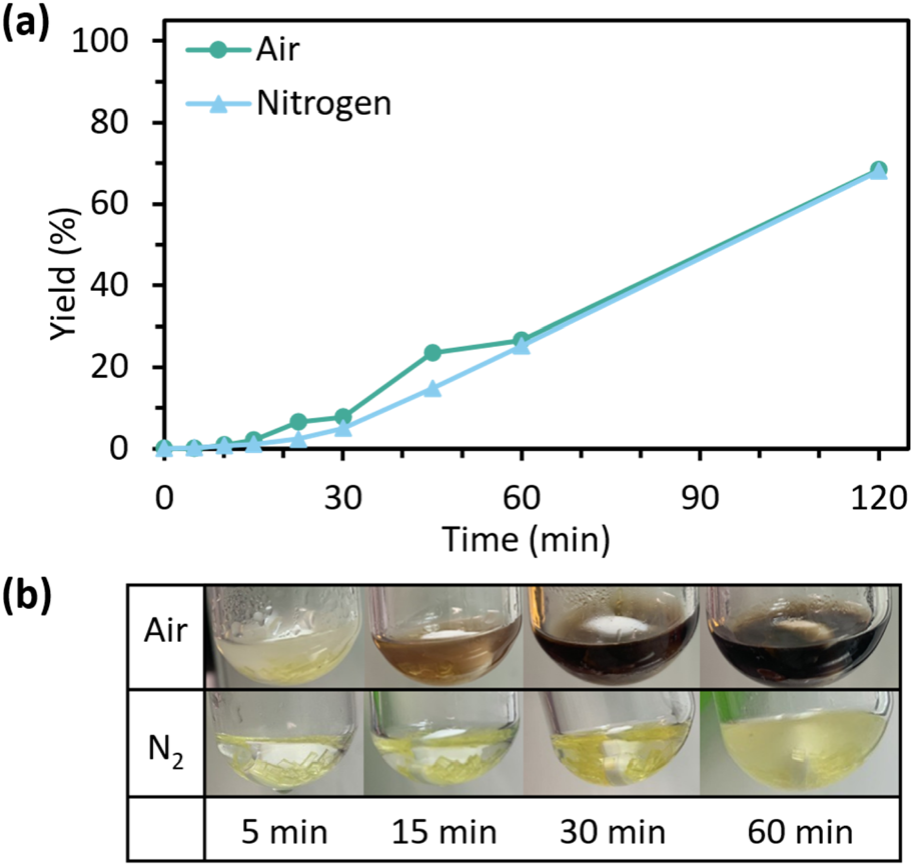
(a) Effect of atmosphere on PET glycolysis, at 5 mol% catalyst, 10 equiv. EG and 180 °C. (b) Colour comparison of glycolysis with air or nitrogen atmosphere (conditions same as Fig. 1c at 5 mol%).

Both, importantly, are non-aromatic and weakly nucleophilic, with TBA being linear aliphatic and pempidine being a cyclic aliphatic amine. The impact of aromaticity was stark at 30 min, with TBA and pempidine only achieving a yield of 3.9% each, compared to 75% for PS (Fig. 5). This difference in reactivity is most visible at early time points, reflecting aromaticity of PS allowing it to intercalate within the PET matrix. TBA and pempidine rates improved from 30 min after swelling and surface depolymerisation disrupted the physical network. The p*K*_a_ in ACN may also be an inadequate measure of basicity under the reaction conditions, as it is both structure and solvent dependent.^28^ In water, the p*K*_a_ of TBA (9.8–10.9)^29,30^ and pempidine (11.3)^31^ are somewhat lower than that of PS (12.0–12.3).^32,33^ Catalyst behaviour in ethylene glycol may fall somewhere between due to its significant hydrogen-bonding capacity. Non-aromatic organocatalysts with a p*K*_a_ in water closer to that of PS were not available for this study. The robustness of PS-catalysed glycolysis of PET, the impact of air and reaction scale were explored (Fig. 6a and S6–S7) as performing reactions under nitrogen adds economic, logistical and environmental burdens. Reactions under air lead to significant discolouration of the reaction (Fig. 6b) however showed little impact on productivity (Fig. 6a). We hypothesize that formation of an intensely coloured species from previously described thermal and thermo-oxidative degradation pathways of amines may be contributing to the colour observed while not affecting catalyst performance over these timescales.^34,35^

The impact of air was more apparent on scaling up the reaction to 10 g of substrate (Fig. S6). Using air and larger, 10 mm^2^ squares of bottle PET produced 6.4 g BHET (48.7%) as tan needle-like crystals (Fig. S7a). The purity of BHET obtained was 98.2% across triplicate measurements by Q-NMR. An improved reaction under air made use of a tubular vessel for better PET-wetting by EG and a 15 min preheat of PET and EG, yielding 8.4 g (64.1%) of BHET with >99% purity after only 2 h at 10 mol% PS-loading (Fig. S7). A portion of the PS catalyst was observed by ^1^H-NMR to be present in the workup filtration residue, along with 17.2% of the theoretical yield of BHET dimer (Table S2).

**Fig. 7:**
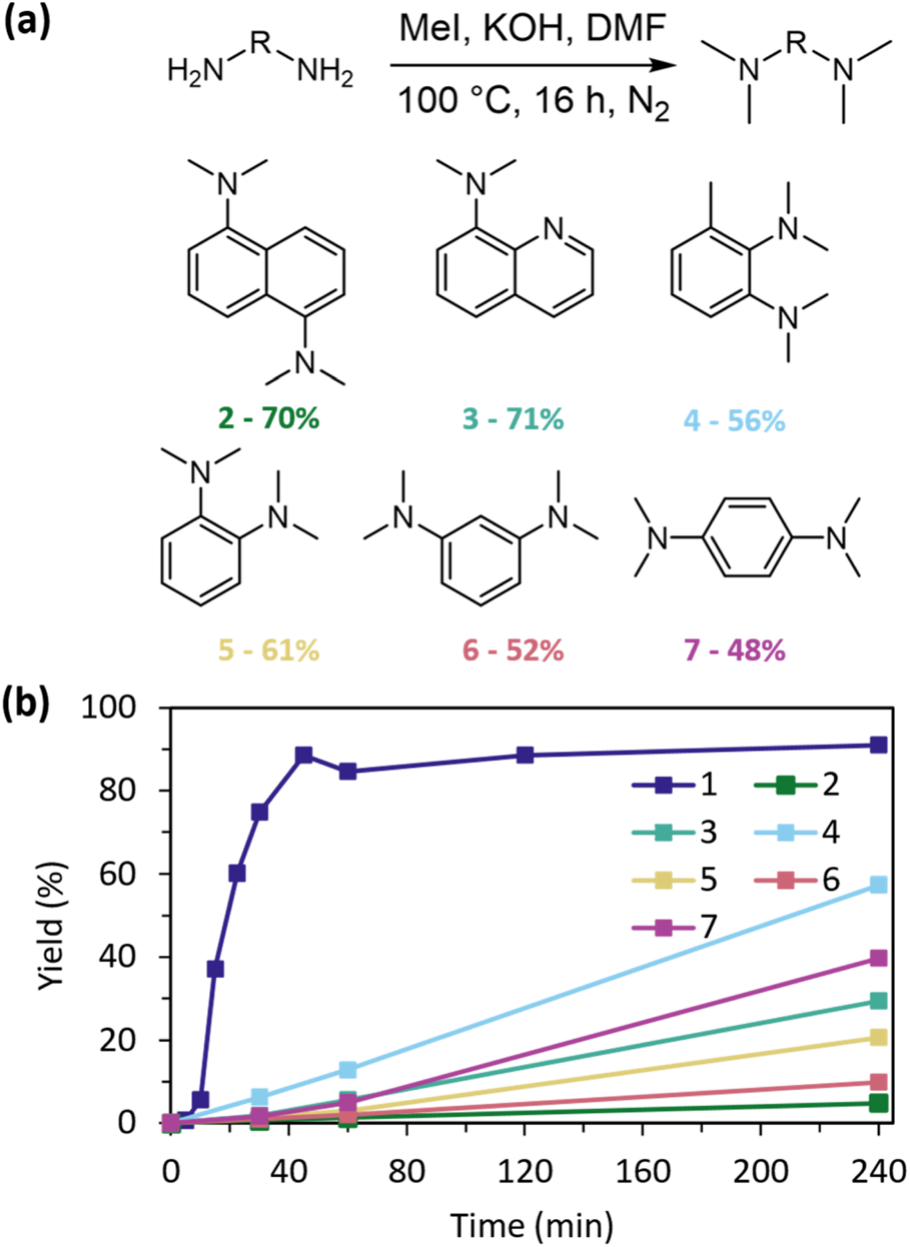
(a) Methylations to produce a library of aromatic amines 2–7 with % yields as determined by ^1^H-NMR spectroscopy. (b) PET glycolysis catalysed by the library of aromatic amines and PS, at 10 equiv. EG, 20 mol% catalyst, 180 °C, N_2_.

To explore the structural uniqueness of PS, we synthesised a range of related aromatic amines to test in glycolysis (Fig. 7a). Initial attempts to produce this library using K_2_CO_3_ as base under the conditions outlined below were unsuccessful, producing minimal amounts of mono-, di-, or tri-methylated products. Switching to a strong base, KOH, gave good yields of each product with structural characterisation by ^1^H-NMR, Heteronuclear Single Quantum Coherence (HSQC), ^13^C-NMR and GC-MS (Fig. S8–S31). This library of methylated aromatic amines was selected to assess effects of changing naphthalene substitution position (2), sp^3^ nitrogen (3), electron-donating and buttressing α-methyl substituent (4), and altered *N*–*N* distances on a benzyl instead of naphthyl skeleton (5–7). The library was tested under standard conditions of 10 equiv. EG, 20 mol% catalyst, 180 °C, and a nitrogen atmosphere (Fig. 7b).

Decreased efficacy across all catalysts supported PS privileged structure. Of variants screened, 4 performed best, highlighting the important effects of the α-methyl group, especially when compared to the performance of 5. This highlights that *N*–*N* proximity alone does not necessarily promote activity. 7, with the maximum *N*–*N* distance on benzyl skeletons, performed 2nd best, due to advantageous resonance structures of *meta*-substituted benzyl diamines. Naphthyl substitution patterns (1,8- *vs.* 1,5- as in 2) and ring structure flexibility to distort upon protonation (as in 3) were important to increasing activity. Across the library of catalysts, p*K*_a_ was only a reasonable predictor of activity at 4 h (*R*^2^ = 0.804) (Table S3) while comparison of p*K*_a_ to activity at earlier timepoints (30 and 60 min) gave no substantive correlation (Fig. S32). Across all catalysts surveyed, activity increased dramatically when p*K*_a_ > 10 (Fig. S32), reiterating the importance of EG deprotonation on the glycolysis mechanism (EG p*K*_a_*ca.* 15 in water).^36^

## Conclusions

Proton Sponge efficiently catalyses the glycolysis of bottle-grade PET into BHET monomer. The aromaticity of PS enhances its catalytic effect when compared to similar strength alkyl amine bases,^19^ with activation energies comparable to other reported catalysts. Interestingly, a pseudo first-order kinetic treatment sufficiently modelled the glycolysis (*R*^2^ > 0.98), suggesting that these aromatic amines may intercalate and overcome surface limitations that previously required more complex modelling, at least for cut bottles as substrates. The catalysis showed an induction period from both thermal and mass-transfer limitations. This could be substantially circumvented by pre-heating PET in EG prior to catalyst addition, with the swelling allowing ingress of the PS quickly into the catalyst structure. 10 equivalents of ethylene glycol provided an optimal trade-off between swelling, rates and dimer production. While the reaction was robust, air does lead to some darkening of the reaction mixture but largely did not affect rate even at larger scales with preheating. A library of methylated aromatic amines suggested the PS structure was particularly privileged as an aromatic amine, with *N*–*N* proximity and buttressing effects most important. We plan on exploring alkyl substitution of PS analogues in future studies to help rationalise observe effects.

While Proton Sponge has shown promise as an organocatalyst, highlighting aromaticity as an interesting avenue for further research, this does not yet suggest commercial relevance for glycolysis. Economic and environmental impacts should be compared across the multiple potential fates of PET to define the role for PET depolymerisation to tackle the expected annual production of 61 million tonnes of PET by 2060.^37^

## Author contributions

R. A. C.: conceptualisation, data curation, formal analysis, funding acquisition, investigation, methodology, writing – original draft, writing – review & editing. C. W. L.: conceptualisation, methodology, writing – review & editing. M. P. S.: conceptualisation, funding acquisition, supervision, writing – review & editing.

## Conflicts of interest

There are no conflicts to declare.

## Supplementary Material

RA-016-D6RA00723F-s001

## Data Availability

The following data are included as part of the supplementary information (SI). Supplementary information: PET characterisation by size, DSC, TGA and GPC; an example ^1^H-NMR spectrum of a crude mixture, and ^1^H-NMR, ^13^C-NMR, HSQC and GC-MS data on the catalyst library. See DOI: https://doi.org/10.1039/d6ra00723f.
