# Supplementary material for: Proton sponge: an aromatic glycolysis catalyst
Source: RSC Adv. 2026 Feb 9;16(9):8044–50. doi: 10.1039/d6ra00723f (PMC12885058; doi:10.1039/d6ra00723f)
Supplement: RA-016-D6RA00723F-s001 [file RA-016-D6RA00723F-s001.pdf]

Supplementary Information (SI) for RSC Advances.
This journal is © The Royal Society of Chemistry 2026

Supplementary Information

Proton Sponge: An Aromatic Glycolysis Catalyst

Robbie A. Clark, Ciaran W. Lahive and Michael P. Shaver

Sustainable Materials Innovation Hub, Henry Royce Institute, University of Manchester, Manchester M13 9BL, United Kingdom

Instrumentation

Nuclear magnetic resonance (NMR) spectra were obtained at 25 °C on either a Bruker AVIII 400 MHz or a Bruker AVIII HD 500 MHz spectrometer. Chemical shifts are given as δ in parts per million (ppm) and are referenced to the residual solvent resonance of DMSO-d_6 (6H δ = 2.500 ppm, 13C δ = 39.520 ppm). Resonance multiplicities are described as s (singlet), d (doublet), t (triplet), q (quartet), or m (multiplet). Thermogravimetric analysis (TGA) was performed on a TA Instruments SDT 650 with alumina pans and a ramp rate of 10 °C/min, differential scanning calorimetry (DSC) on a TA instruments DSC2500 with a ramp rate of 10 °C/min using sealed aluminium pans, under N_2 atmosphere. Gel permeation chromatography (GPC) was carried out on an Agilent 1260 Infinity II LC system with two Mixed-C Columns (PLGel 5 µM, 300 x 7.5 mm). A HPLC-grade chloroform mobile phase and a polystyrene standard-based conventional calibration underpinned the triple detection by light scattering, refractive index and viscometry detectors. PET was dissolved for GPC by addition of a 1:20 volume ratio solution of trifluoracetic acid in chloroform with stirring for ~10 min, followed by filtration through a 0.2 µm polytetrafluoroethylene (PTFE) filter. High resolution gas chromatography-mass spectrometry (GC-MS) was carried out using a Shimadzu GC 2010 Plus coupled with a QP2010 SE Gas Chromatography Mass Spectrometer, using electrospray ionisation (ESI) on an Agilent QTOF 6530 coupled with HPLC Infinity II.

Poly(ethylene terephthalate) Characterisation

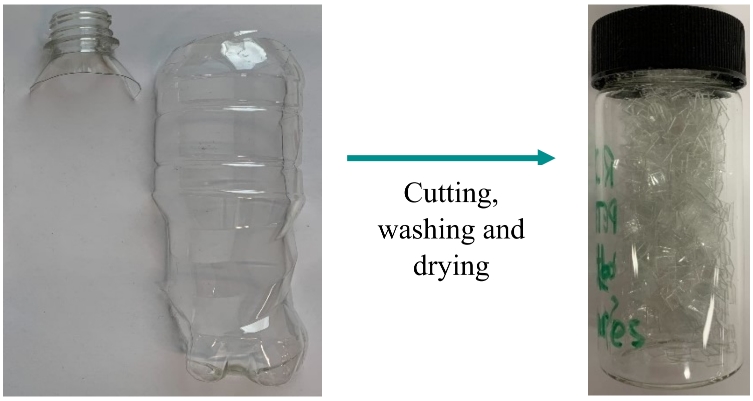

Fig. S1 Pre-consumer, colourless PET bottles used in this study.

To measure the size of the PET squares used, two sets of squares (15 and 25 squares respectively) were taken from different instances of cutting and measured (length, width, thickness) with a micrometer.

Table S1 The average dimensions and mass of PET squares.

	Length (mm)	Width (mm)	Thickness (mm)	Mass (mg)	Surface area (mm^2)
1st set	4.82	3.24	0.31	5.78	36.32

2nd set	3.09	3.24	0.30	3.33	22.95
Overall	3.74	3.24	0.30	4.25	27.96
Standard deviation	1.32	0.90	0.03	1.87	10.67

Differential scanning calorimetry

Glass-transition temperature (T_g), melting temperature (T_m) and crystallinity (X_c) were calculated using the in-built tools in TRIOS v5.1.1.46572. Crystallinity was calculated using the following formula:

$$X_c = \frac{(\Delta H_m - \Delta H_c)}{\Delta H_m{}^{\circ}} \times 100$$

Where X_c is crystallinity (%), ΔH_m is the enthalpy of melting (J/g), ΔH_c is the enthalpy of crystallisation (J/g) and $\Delta H_m{}^0$ is the enthalpy of fusion for 100% crystalline PET (140.1 J/g).

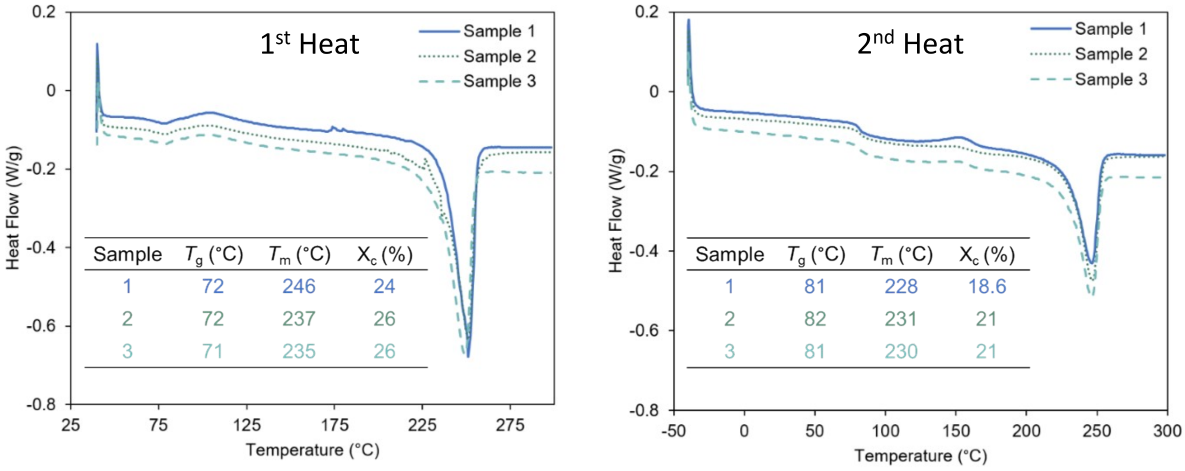

Fig. S2 PET DSC thermograms across the first and second heat.

Thermogravimetric analysis

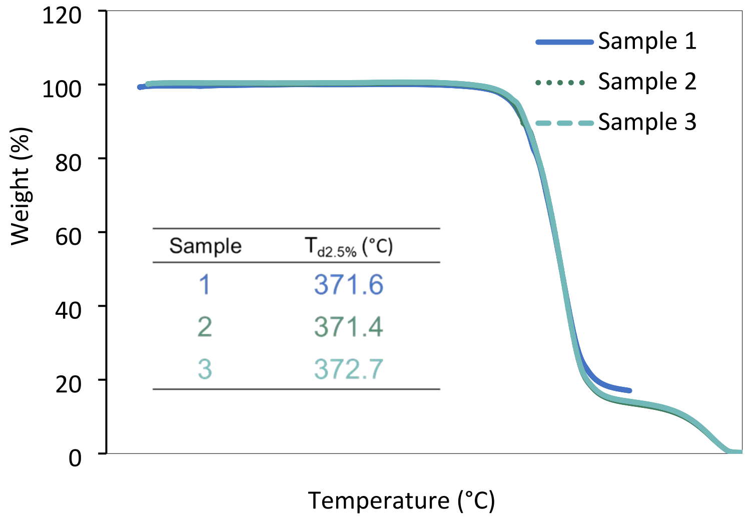

Fig. S3 PET TGA thermograms across three samples under nitrogen at 100 ml/min flowrate, measured at a 10 °C/min ramp rate. Temperature at 2.5% mass loss ($T_{d2.5\%}$) shown.

Gel-permeation chromatography

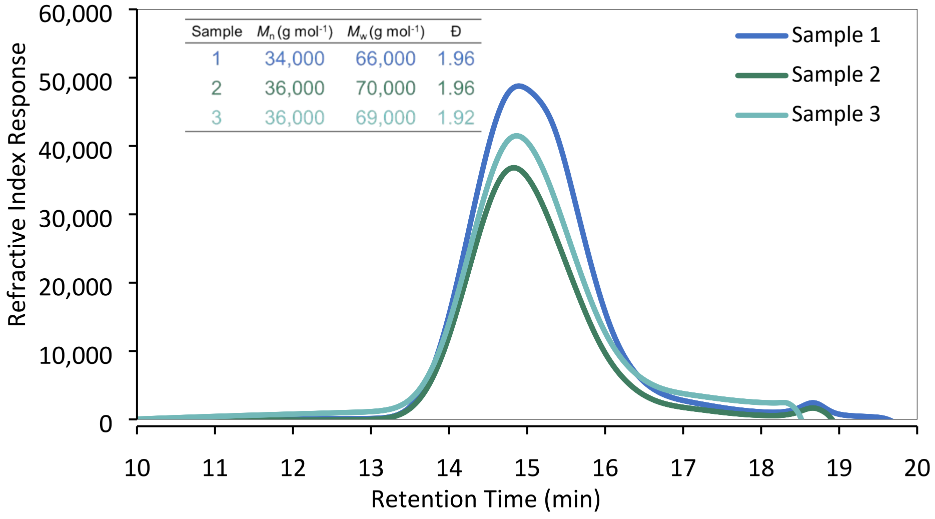

Fig. S4 PET GPC traces across three samples.

Quantitative NMR (Q-NMR)

To calculate the yield of BHET by Q-NMR, the integral of a BHET peak (m, 4H, 8.03-8.17 ppm) was compared to the integral of a diphenyl ether internal standard peak (t, 2H, 7.13 ppm) (Fig. **S5**), along with the moles of diphenyl ether within the NMR sample, by the following equation:

$$mol_{BHET} = \frac{int_{BHET}}{int_{DPE}} \times \frac{N_{DPE}}{N_{BHET}} \times mol_{DPE}$$

Where mol_{BHET} = moles of BHET in the NMR sample, int_{BHET} = integral of the BHET peak, int_{DPE} = integral of the diphenyl ether peak, N_{DPE} = number of protons of the DPE peak, N_{BHET} = number of protons of the BHET peak and mol_{DPE} = moles of diphenyl ether in the NMR sample.

The above assumptions and calculations are complicated by the presence of dimeric BHET, and possibly trimeric BHET as glycolysis products. These products are evidenced by two means: first, the BHET aromatic peak is not a clean singlet, as would be expected with monomeric BHET, but rather has a small quartet and other peaks adjacent which represent the aromatic protons of BHET dimers and/or trimers. Second, a small peak at 4.67 ppm is the internal ethylene glycol linker within a dimer, with this value being in accordance with literature.[1] The presence of trimer has previously been linked to a peak 4.7 ppm[2], however this was not visible in our samples and may have been included by the dimer peak. The poor-solubility of PET trimer in DMSO may be responsible for this absence. Therefore, to calculate the yield of monomeric BHET, the multiplet peak at 8.05-8.17 ppm was used to determine the amount of BHET in either form (with an assumed proton correspondence of 4), and the peak at 4.67 ppm was used (with a proton correspondence of 4) to determine the amount of BHET dimer, and then subtract this amount off of the 8.05-8.17 value to give the monomeric BHET yield.

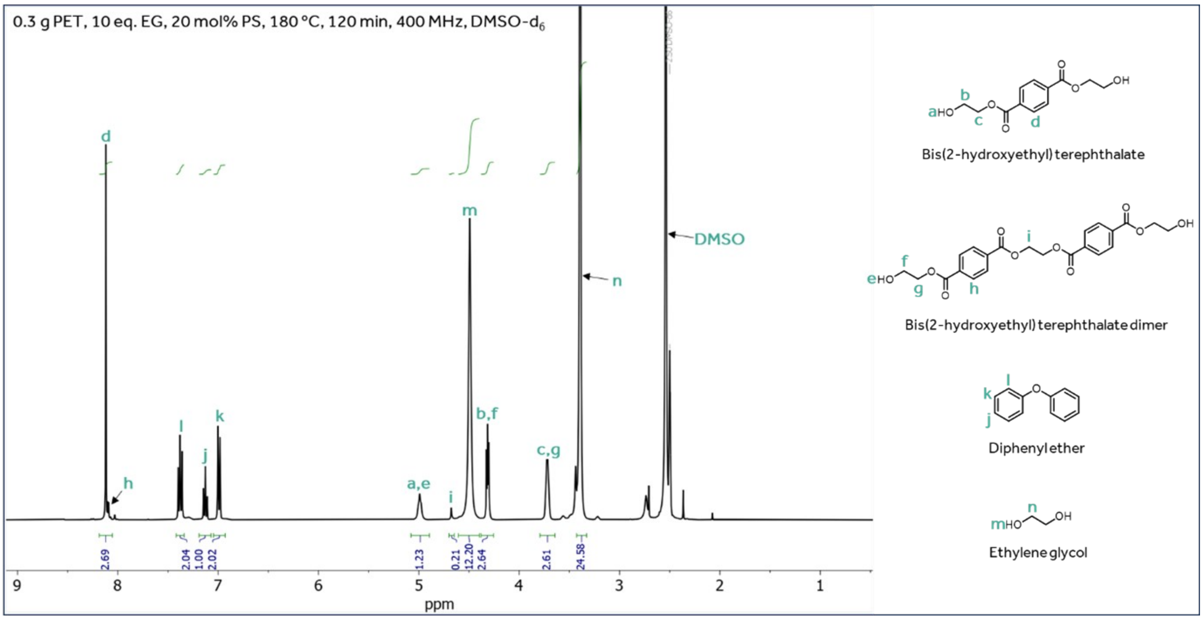

Fig. S5 Example ^{1}H-NMR spectrum of the products of PS-catalysed glycolysis.

Ten-gram scale glycolysis

PET (10 g), EG (32.3 g, 10 equiv.), PS (1.12 g, 10 mol%) and a stirrer bar were added to a 500 ml round-bottomed flask fitted with a waterless condenser, or to a Schlenk flask (**Fig. S7**). The flask was lowered into a pre-heated oil bath (190 °C) and stirred (250 rpm) for a desired time until all visible PET pieces had dissolved. The flask was raised, and ~100 ml of 80 °C deionised water was added to the flask. The flask was stirred for ~2 min, before the mixture being filtered through a glass sinter funnel (preheated to 80 °C) under vacuum. Within the Büchner flask, the filtrate (clear brown liquid, **Fig. S7**) was covered with foil and clamped in a raised position above the fume-hood floor, to allow air-cooling to room temperature, before being placed in a 2 °C fridge for 24 h. Small off-white-to-brown crystals were then collected by vacuum filtration through a sintered glass funnel. The BHET crystals were dried to a constant mass in a 60 °C vacuum oven, taking 24 h. Three samples (~0.02 g) of the crystal were dissolved in a DMSO-d_6 stock solution and analysed by the standard QNMR method. A yield and mass purity of BHET and dimer was calculated (**Table S2**).

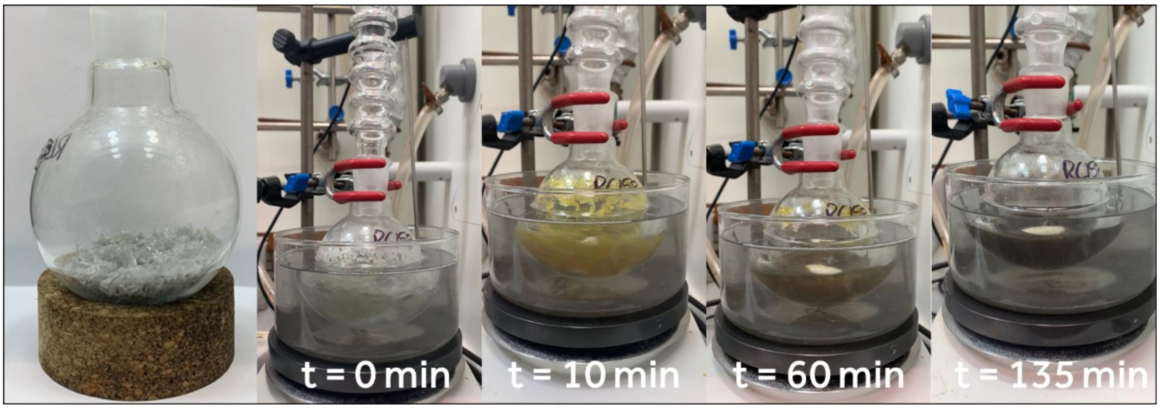

Fig. S6 visual change to BHET across large scale reaction.

The larger scale reactions shown below were carried out in a round-bottomed flask (first run, **Fig. S7a**) and a Schlenk flask (second run, **Fig. S7b**). The Schlenk flask's shape was more beneficial as fewer

PET flakes would be kicked up by the stirrer bar onto the wall of the flask, when compared to the round-bottomed flask.

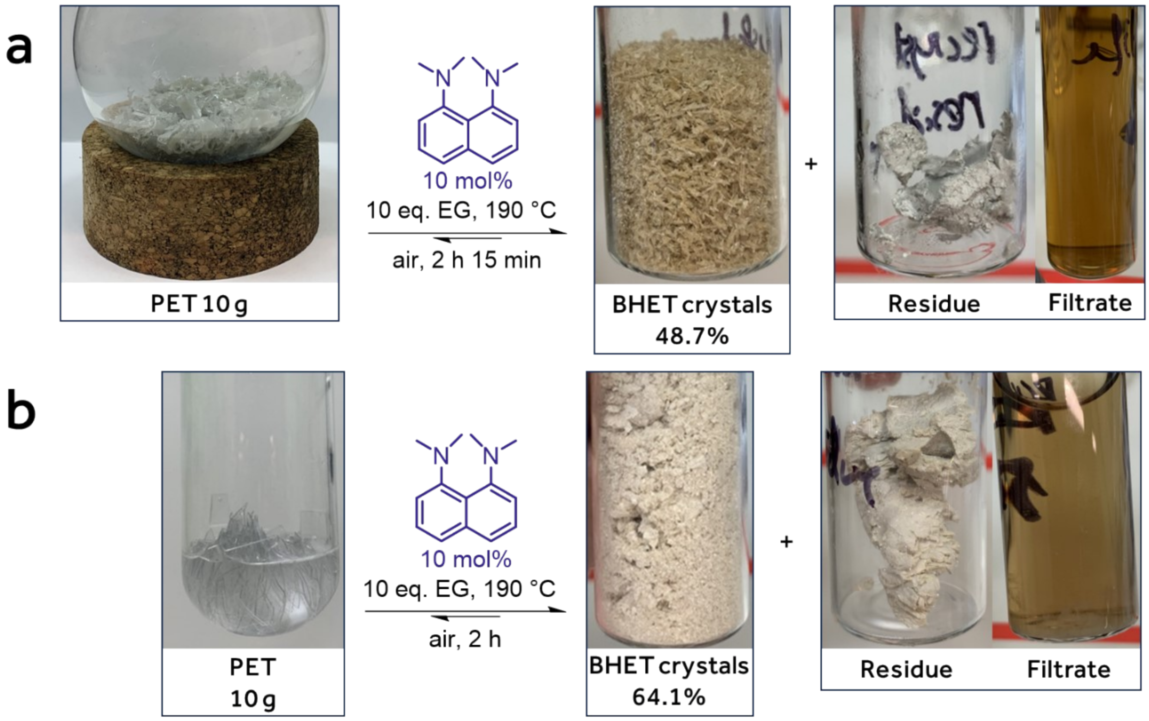

Fig. S7 a Summary of conditions and products of the first run of the 10 g PET glycolysis by PS. The residue and filtrate were collected during hot filtration of the reaction mixture with the added 100 ml 80 °C water to allow BHET crystallisation **b** Conditions and products from the second run of the same reaction.

Table S2 Summary of the BHET content of each fraction of each run, and other major constituents. Measured by QNMR.

Run	Fraction	Mass (g)	BHET content (mass%)	Of BHET total (mol%)	Other constituents (mass%)
first	BHET Crystals	6.44	98.2	47.8	-
	Residue	1.37	32.8	3.4	BHET dimer (28.6)
	Filtrate	-	-	6.7	-
second	BHET Crystals	8.41	100.0	63.6	-
	Residue	2.58	26.2	8.6	dimer (18.3) + Proton Sponge (17.2)
	Filtrate	-	-	14.5	-

N1,N1,N5,N5-tetramethylnaphthalene-1,5-diamine (TMN-1,5)

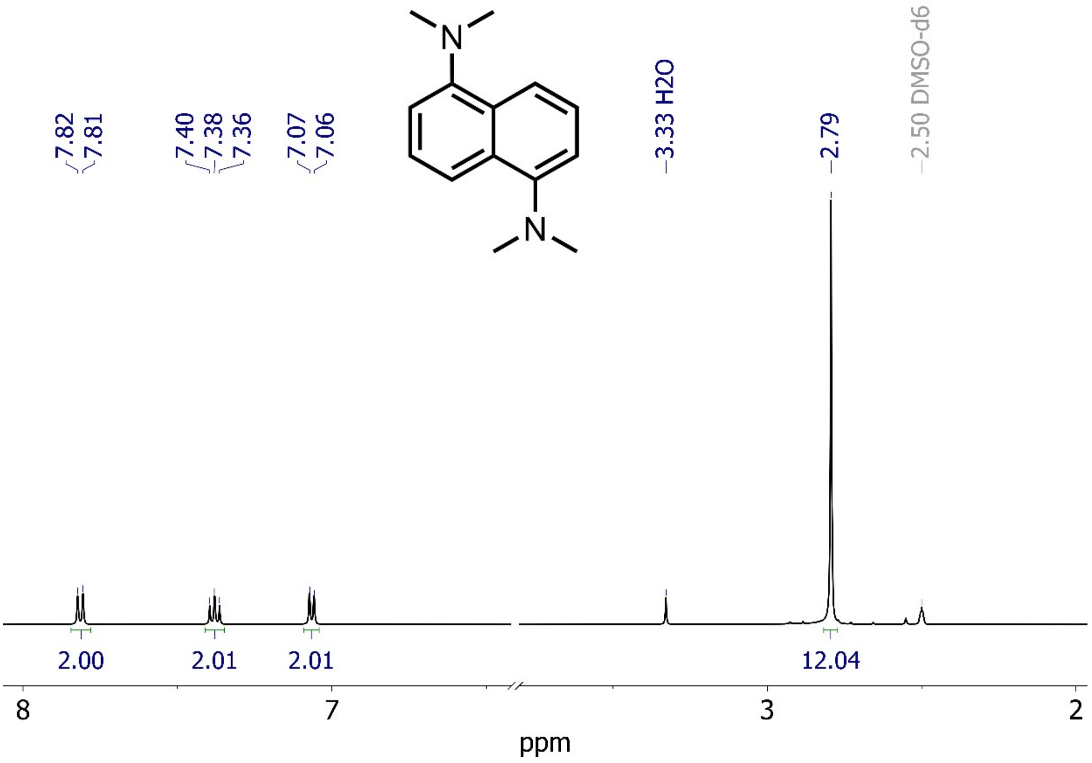

Fig. S8 ^{1}H-NMR spectrum of catalyst TMN-1,5. (500 MHz, DMSO) δ 7.81 (d, *J* = 8.7 Hz, 2H), 7.41–7.35 (t, 2H), 7.06 (d, *J* = 7.5 Hz, 2H), 2.79 (s, 12H).

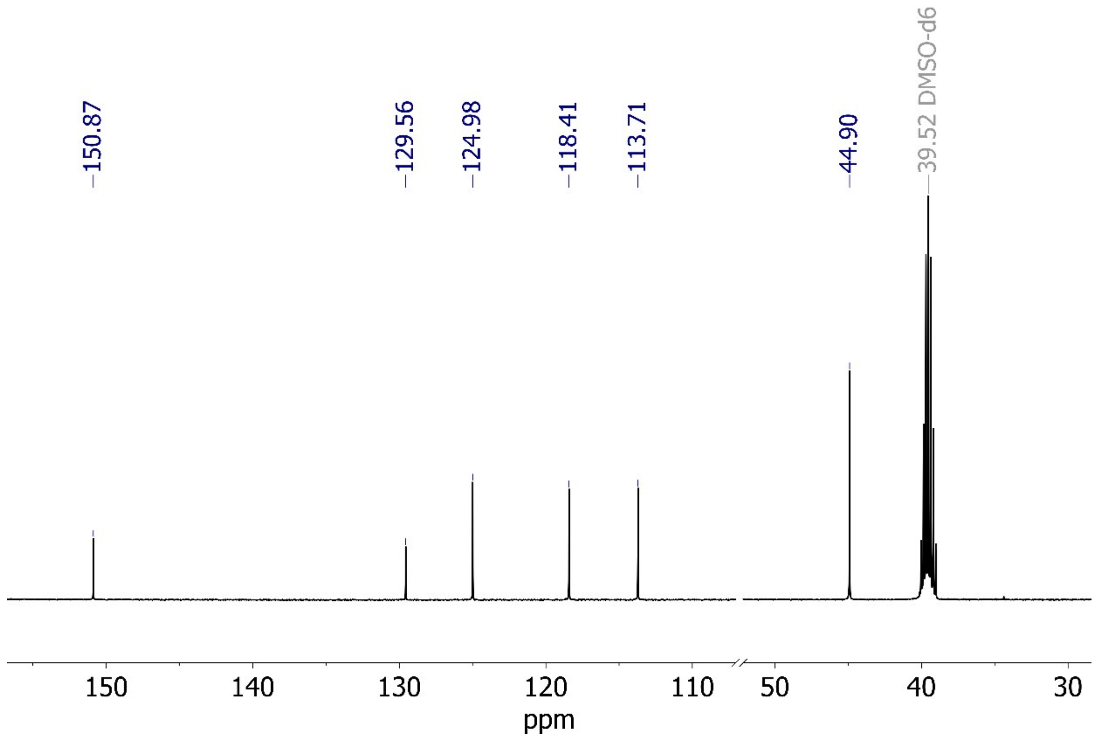

Fig. S9 ^{13}C-NMR spectrum of catalyst TMN-1,5. (126 MHz, DMSO) δ 150.87, 129.56, 124.98, 118.41, 113.71, 44.90.

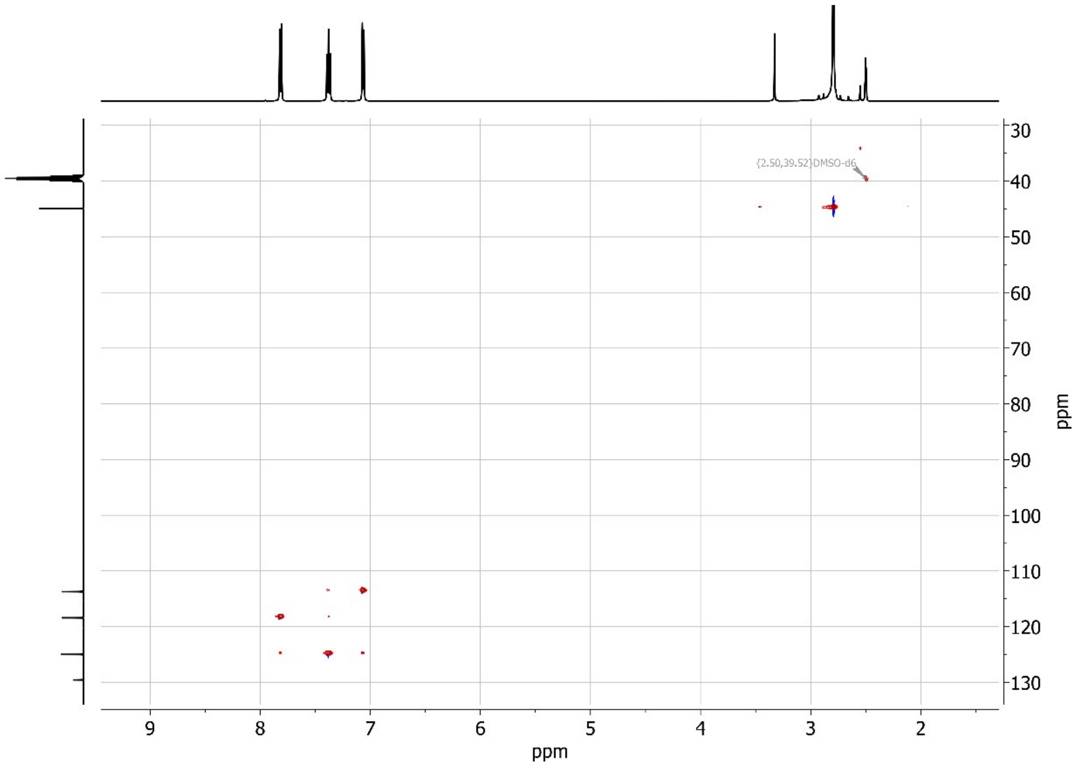

Fig. S10 HSQC spectrum of catalyst TMN-1,5.

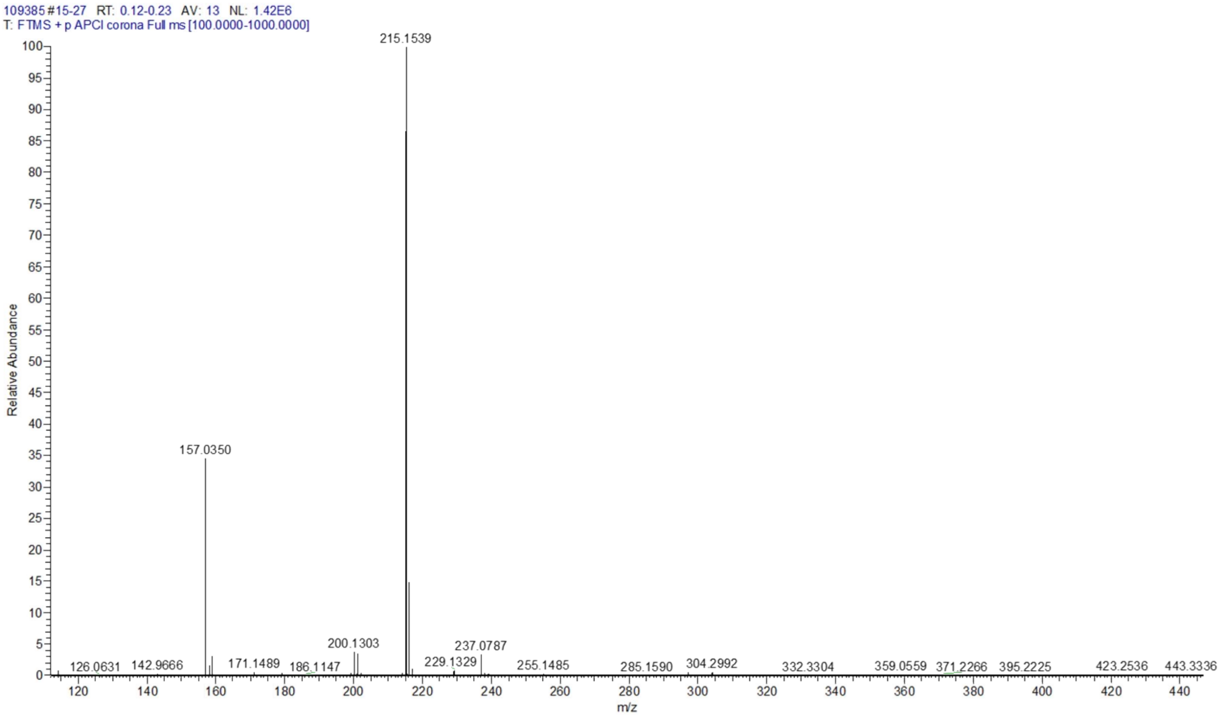

Fig. S11 GC-MS spectrum of TMN-1,5 obtained by electrospray atmospheric pressure chemical ionisation (APCI). Target m/z = 214.15.

N,N-dimethylquinolin-8-amine (DMQ-8)

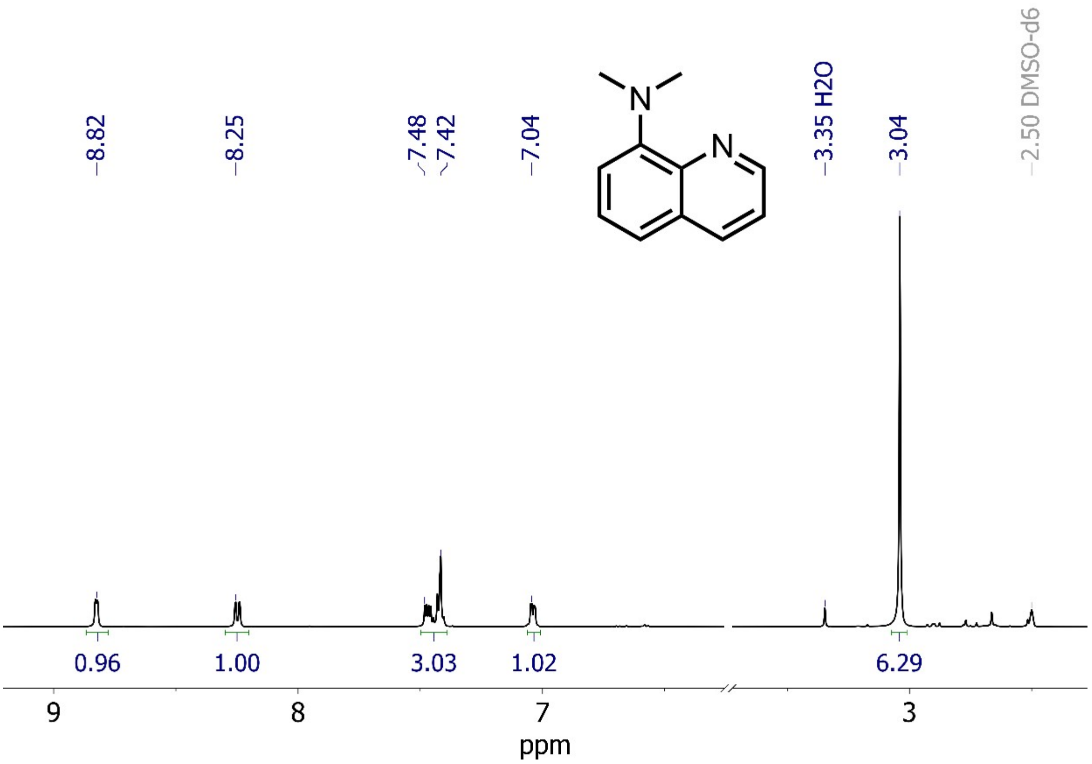

Fig. S12 ^{1}H-NMR spectrum of catalyst DMQ-8. (500 MHz, DMSO) δ 8.82 (d, *J* = 4.1 Hz, 1H), 8.25 (d, *J* = 8.2 Hz, 1H), 7.50 – 7.40 (m, 3H), 7.04 (d, *J* = 6.6 Hz, 1H), 3.04 (s, 6H).

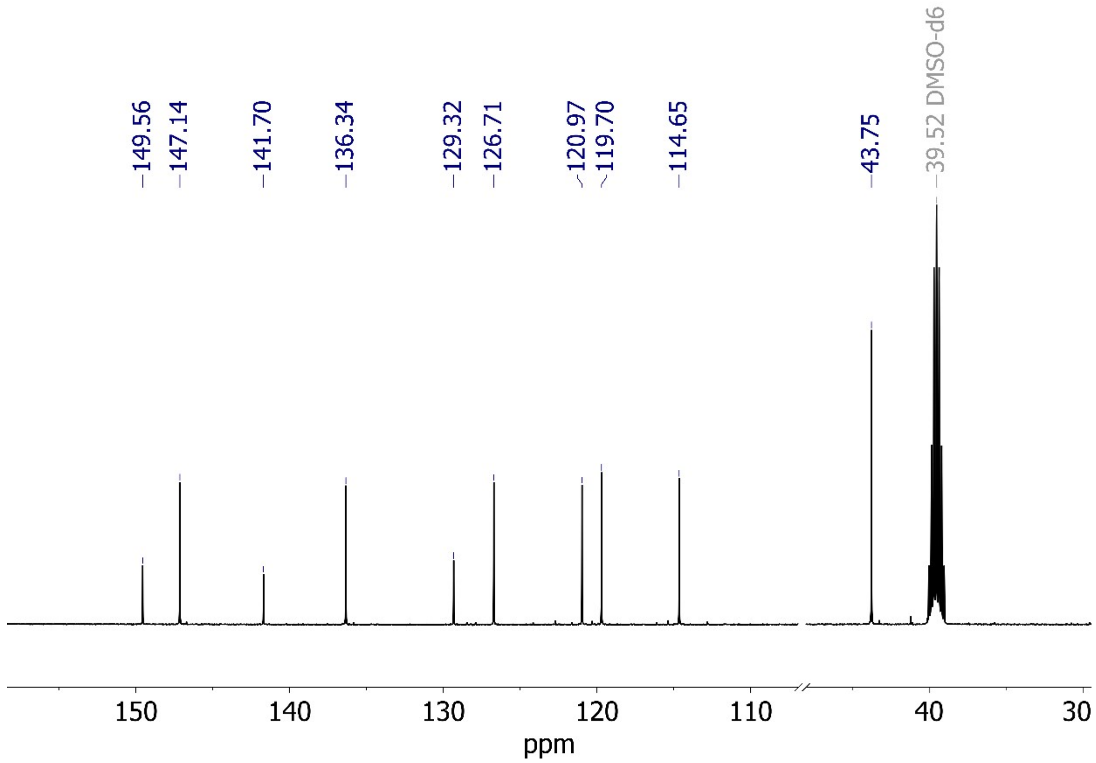

Fig. S13 ^{13}C-NMR spectrum of catalyst DMQ-8. (126 MHz, DMSO) δ 149.56, 147.14, 141.70, 136.34, 129.32, 126.71, 120.97, 119.70, 114.65, 43.75.

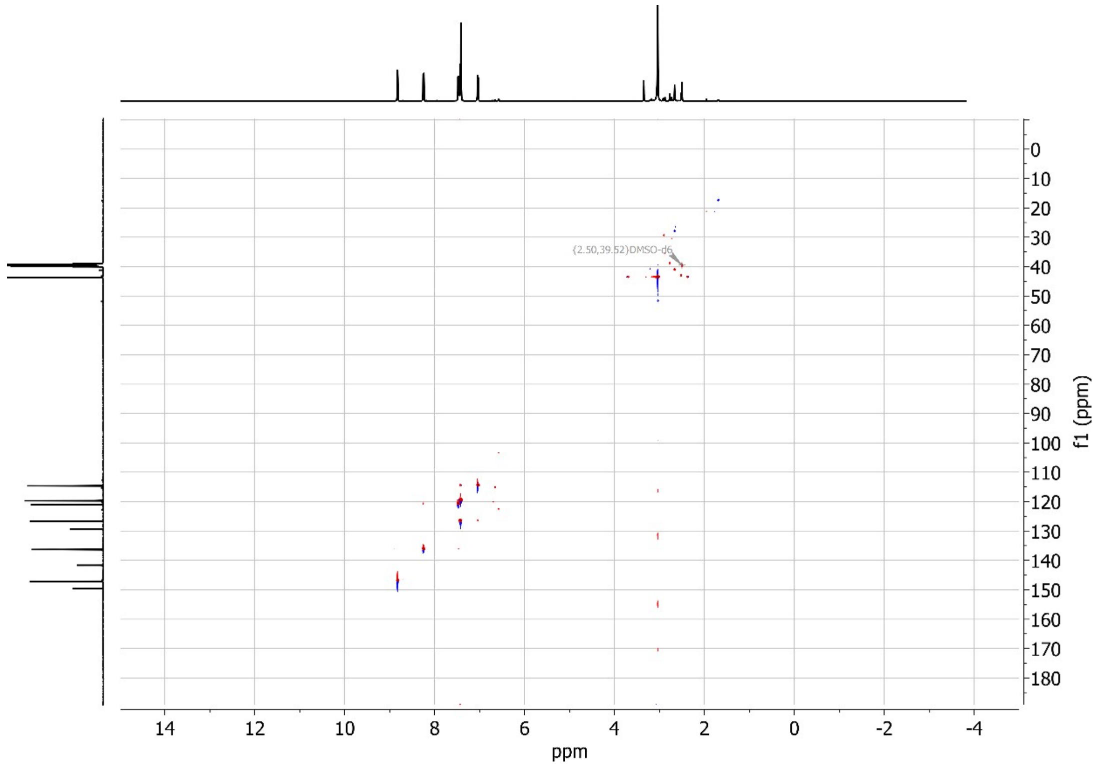

Fig. S14 HSQC NMR spectrum of catalyst DMQ-8.

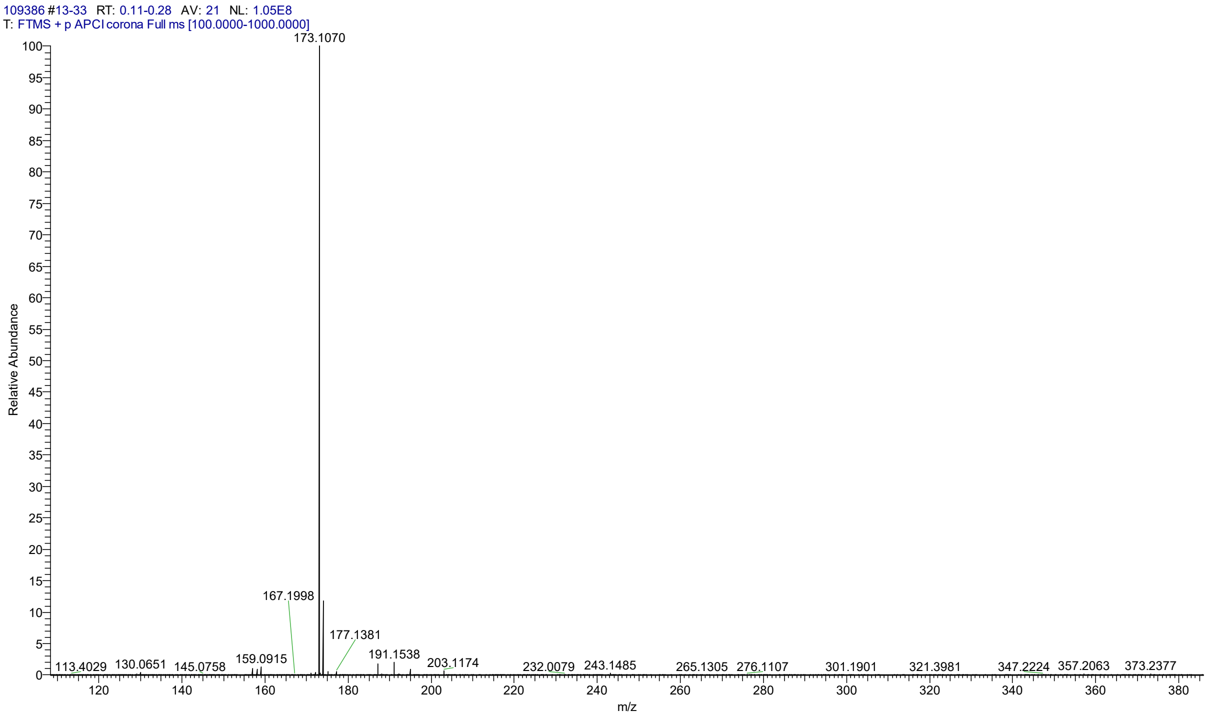

Fig. S15 GC-MS spectrum of DMQ-8 obtained by electrospray APCI. Target m/z = 172.10.

N1,N1,N2,N2,3-pentamethylbenzene-1,2-diamine (3-PMB-1,2)

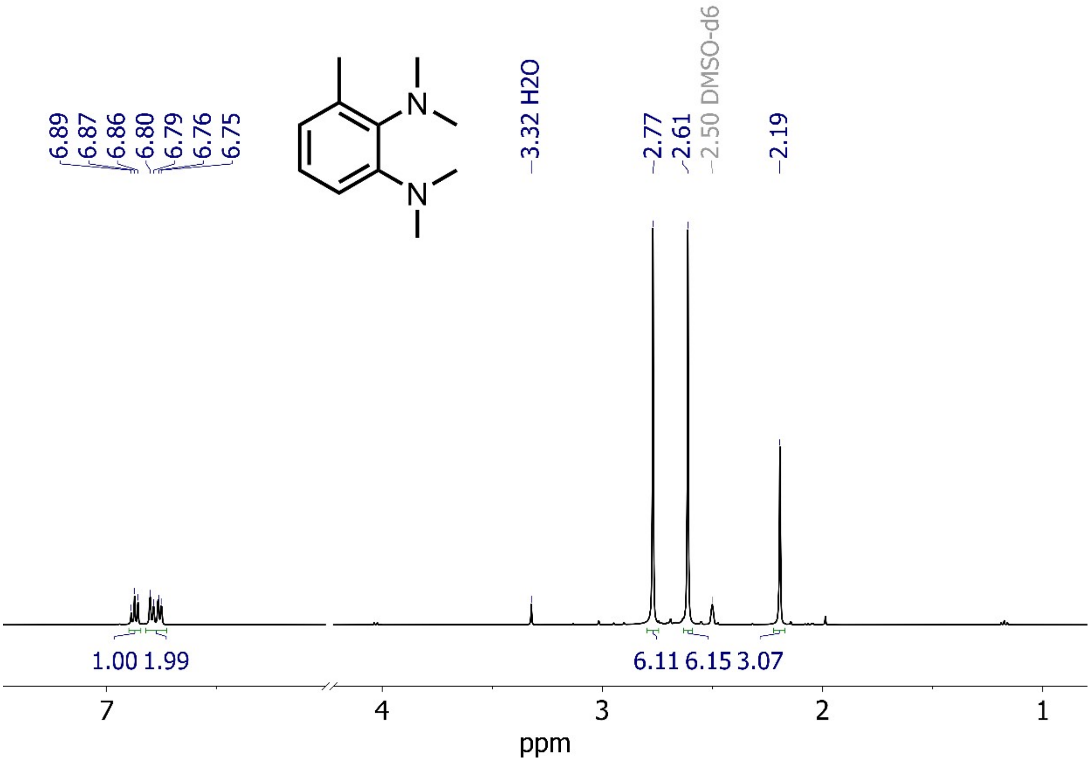

Fig. S16 1H-NMR spectrum of catalyst 3-PMB-1,2. (500 MHz, DMSO) δ 6.87 (t, *J* = 7.7 Hz, 1H), 6.78 (dd, *J* = 18.0, 7.7, 1.7 Hz, 2H), 2.77 (s, 3H), 2.61 (s, 3H), 2.19 (s, 3H).

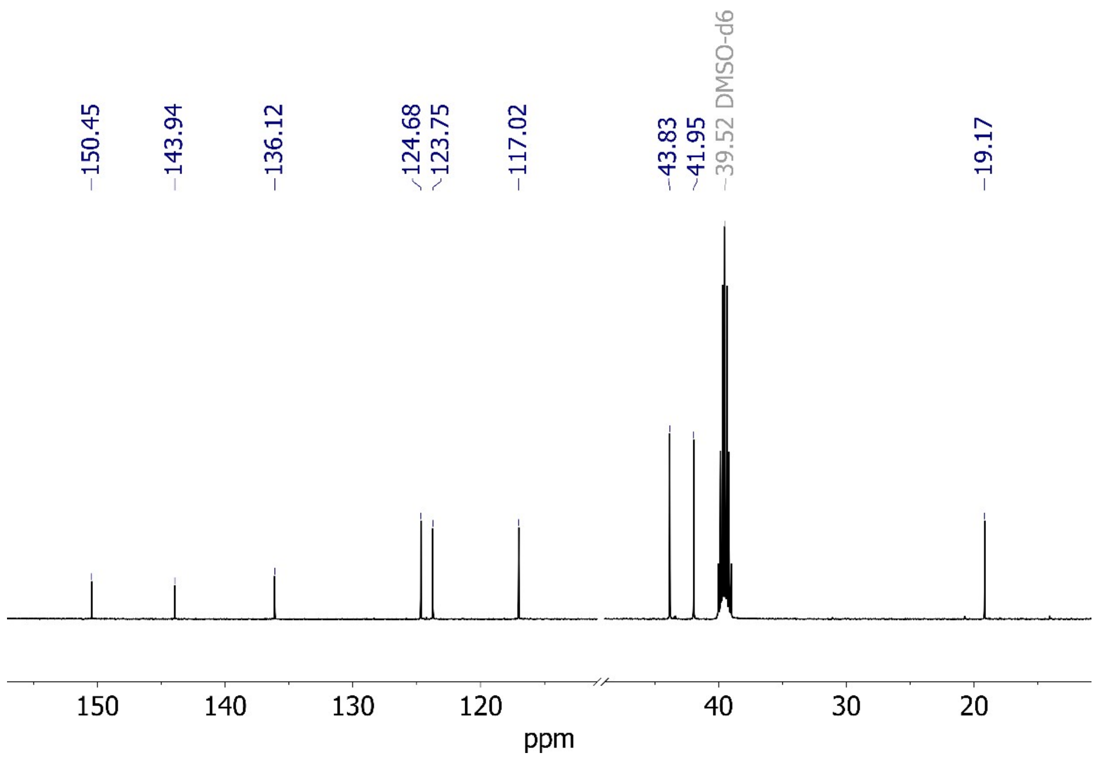

Fig. S17 ^{13}C-NMR spectrum of catalyst 3-PMB-1,2. (126 MHz, DMSO) δ 150.45, 143.94, 136.12, 124.68, 123.75, 117.02, 43.83, 41.95, 19.17.

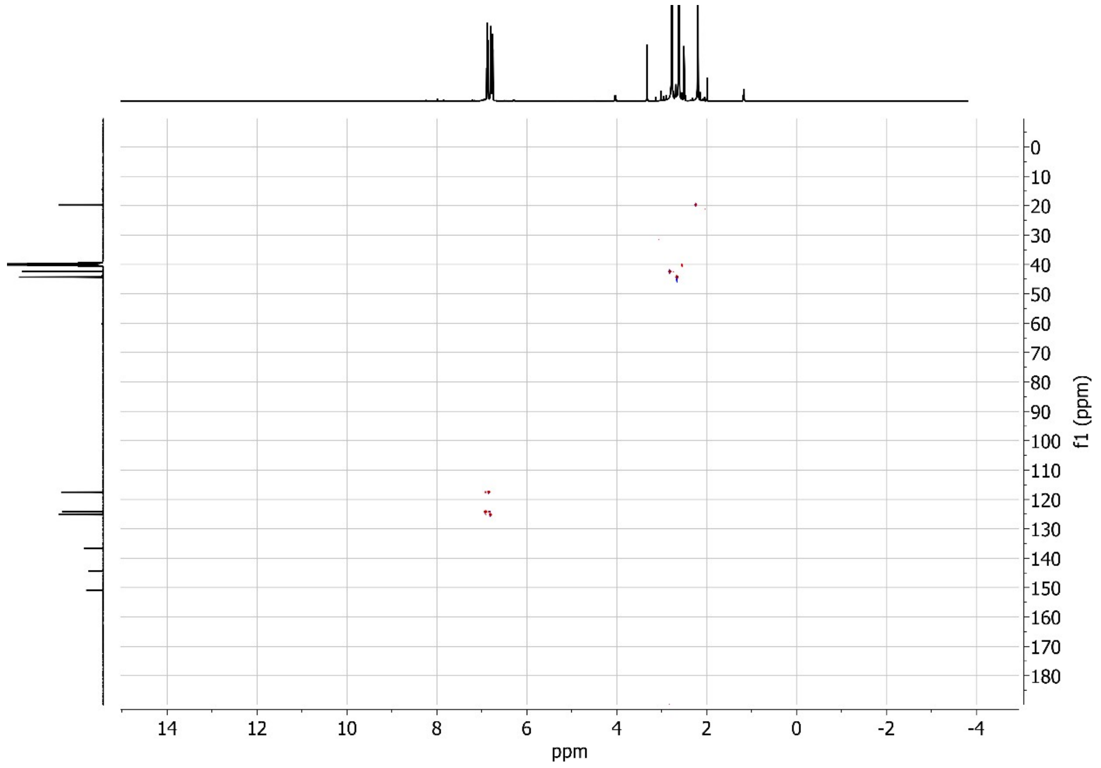

Fig. S18 HSQC NMR spectrum of catalyst 3-PMB-1,2.

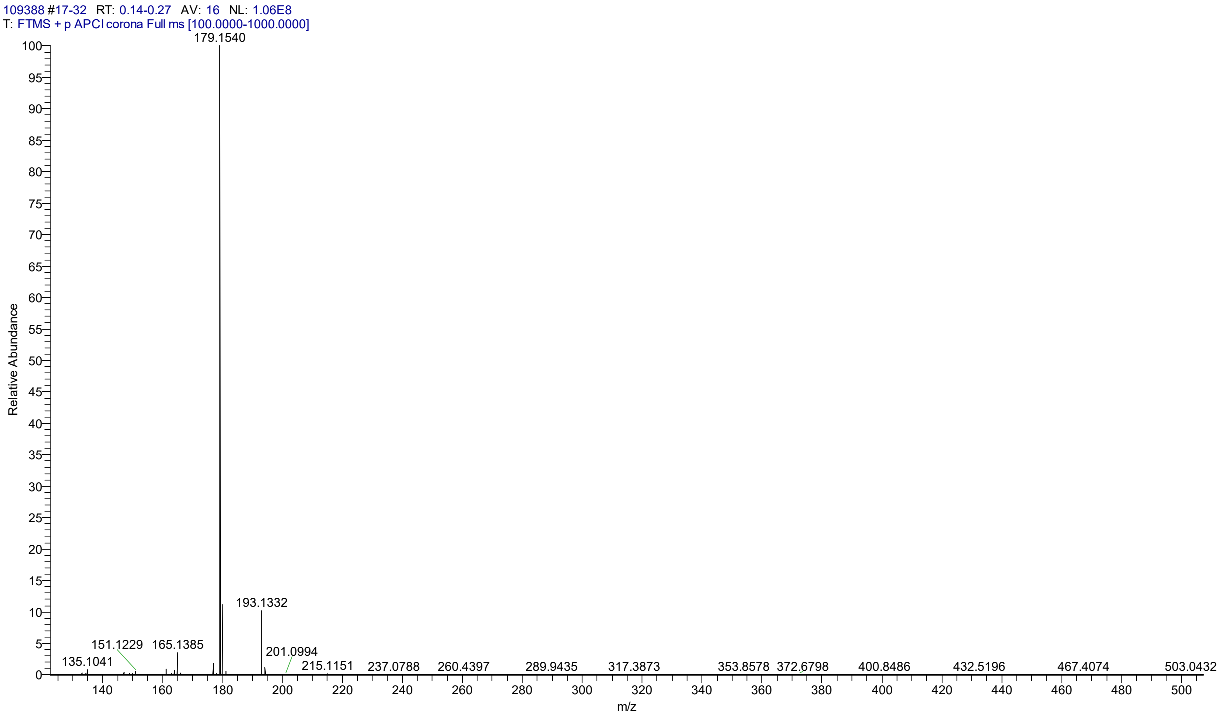

Fig. S19 GC-MS spectrum of 3-PMB-1,2 obtained by electrospray APCI. Target m/z = 178.15.

N1,N1,N2,N2-tetramethylbenzene-1,2-diamine (TMB-1,2)

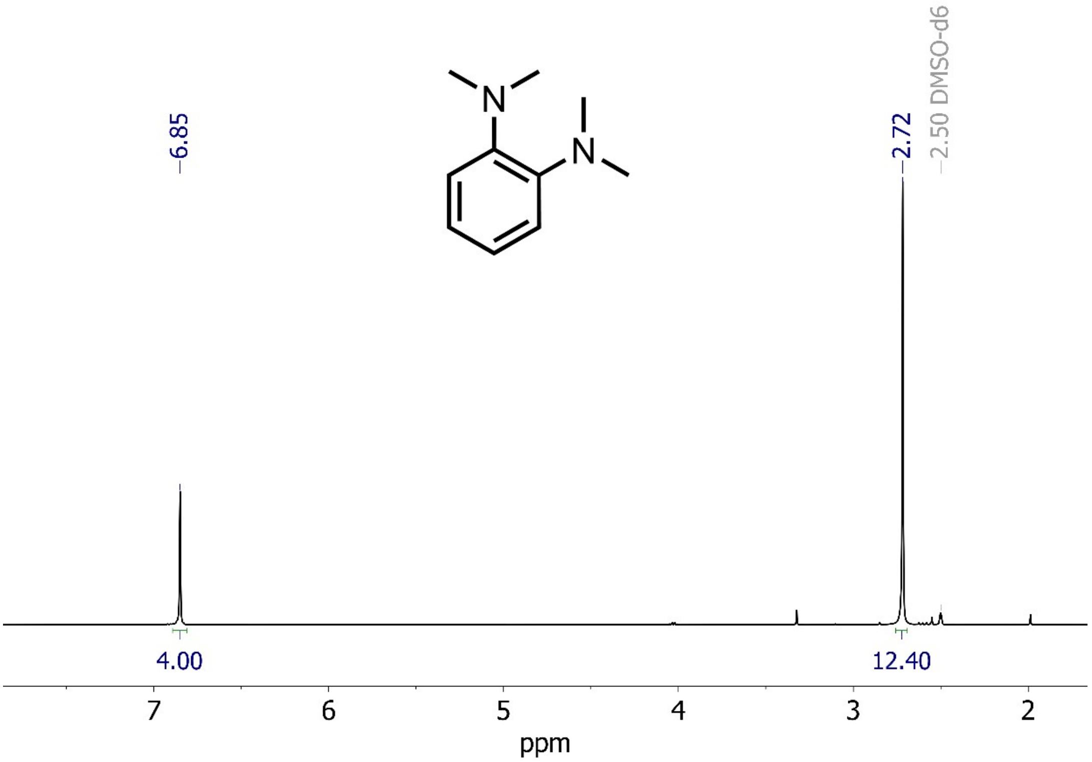

Fig. S20 ^{1}H-NMR spectrum of catalyst TMB-1,2. (500 MHz, DMSO) δ 6.85 (s, 4H), 2.72 (s, 12H).

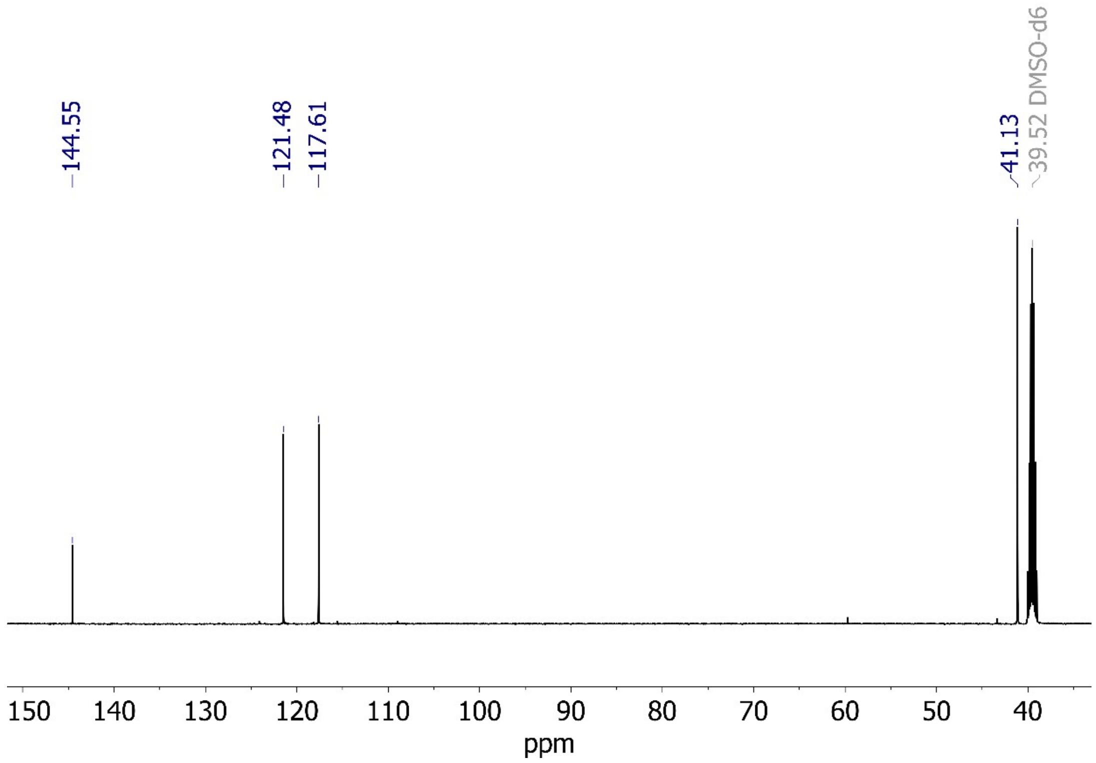

Fig. S21 ^{13}C-NMR spectrum of catalyst TMB-1,2. (126 MHz, DMSO) δ 144.55, 121.48, 117.61, 41.13.

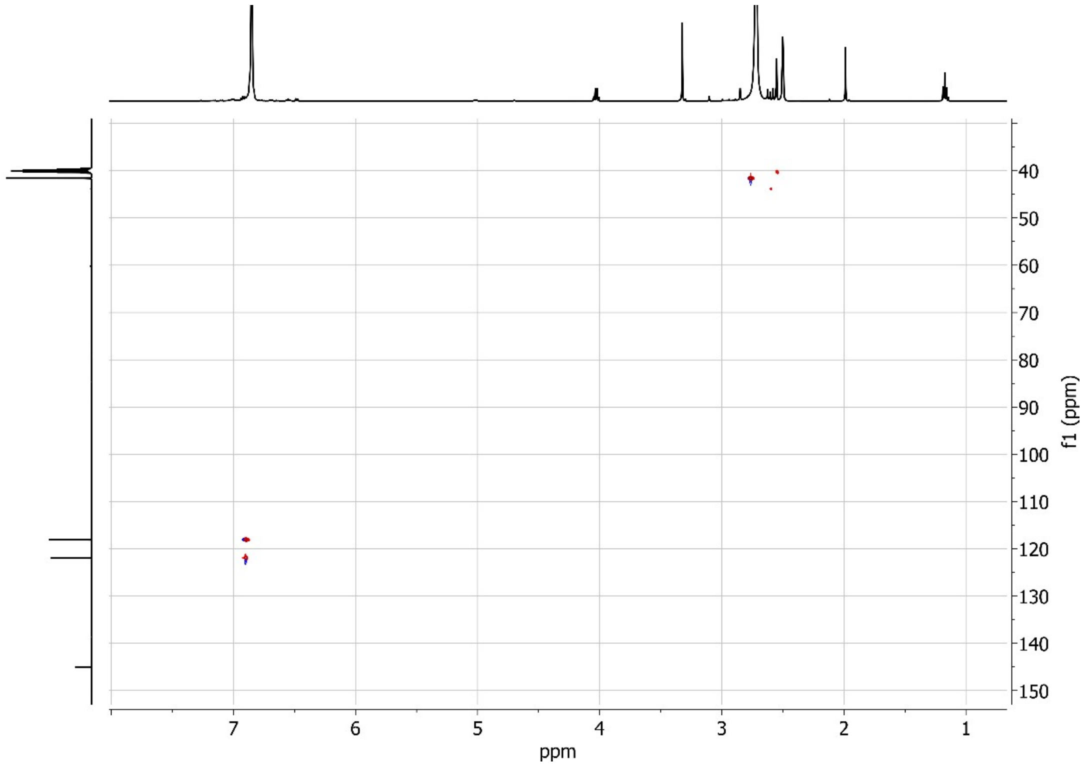

Fig. S22 HSQC NMR spectrum of catalyst TMB-1,2.

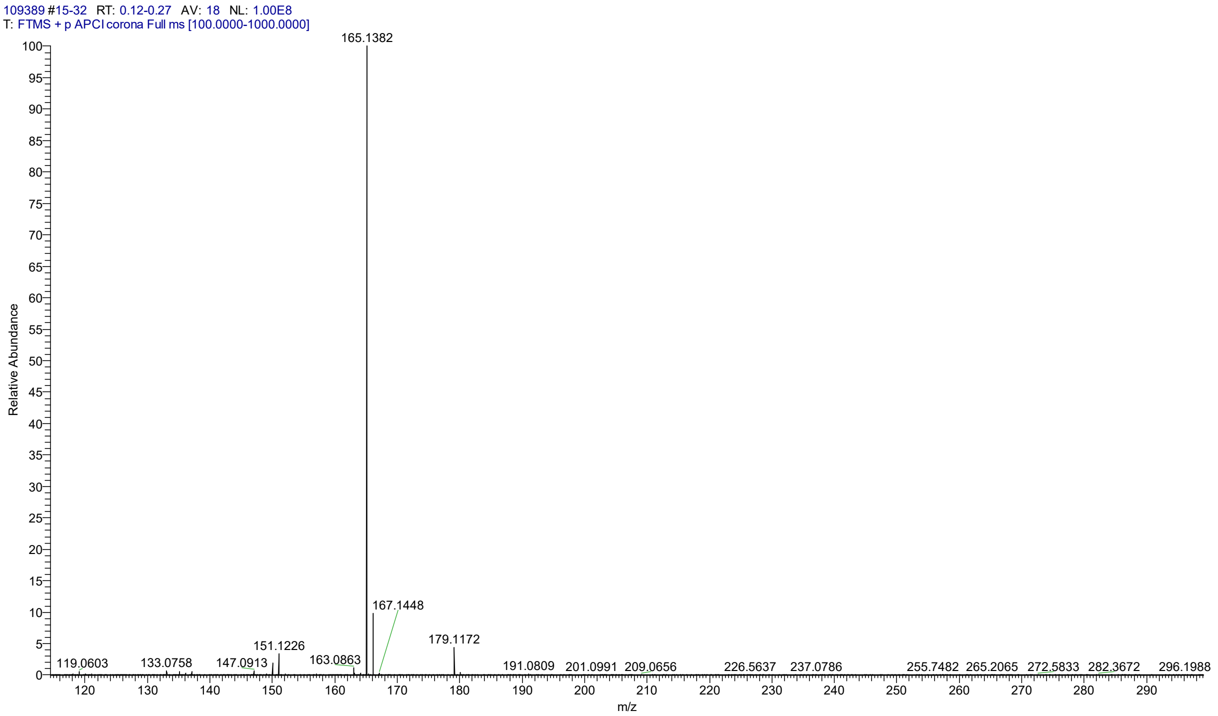

Fig. S23 GC-MS spectrum of TMB-1,2 obtained by electrospray APCI. Target m/z = 164.13.

N1,N1,N3,N3-tetramethylbenzene-1,3-diamine (TMB-1,3)

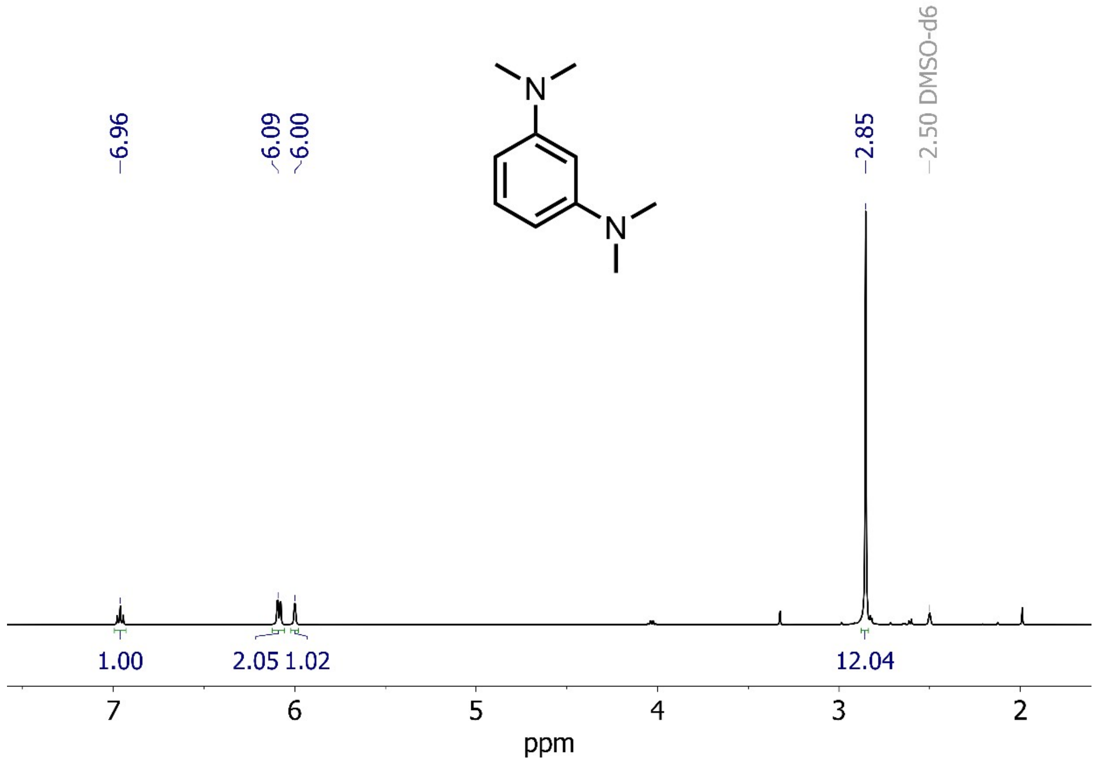

Fig. S24 ^{1}H-NMR spectrum of catalyst TMB-1,3. (500 MHz, DMSO) δ 6.96 (t, *J* = 8.2 Hz, 1H), 6.09 (dd, *J* = 8.1, 2.3 Hz, 2H), 6.00 (t, *J* = 2.3 Hz, 1H), 2.85 (s, 12H).

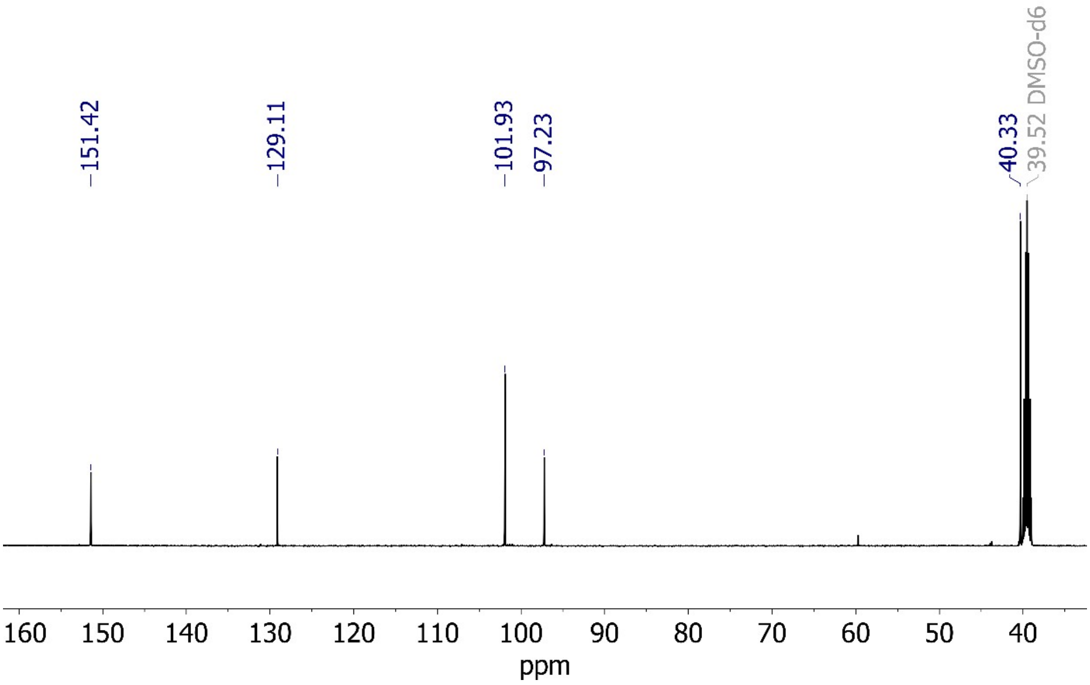

Fig. S25 ^{13}C-NMR spectrum of catalyst TMB-1,3. (126 MHz, DMSO) δ 151.42, 129.11, 101.93, 97.23, 40.33.

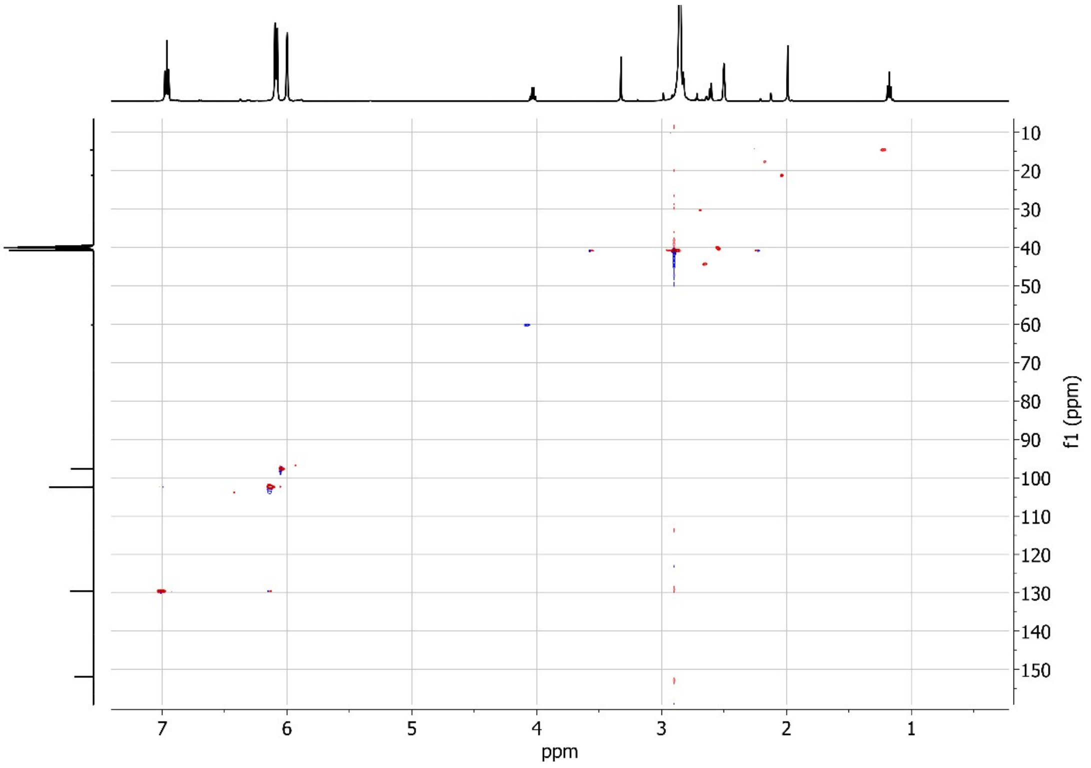

Fig. S26 HSQC NMR spectrum of catalyst TMB-1,3.

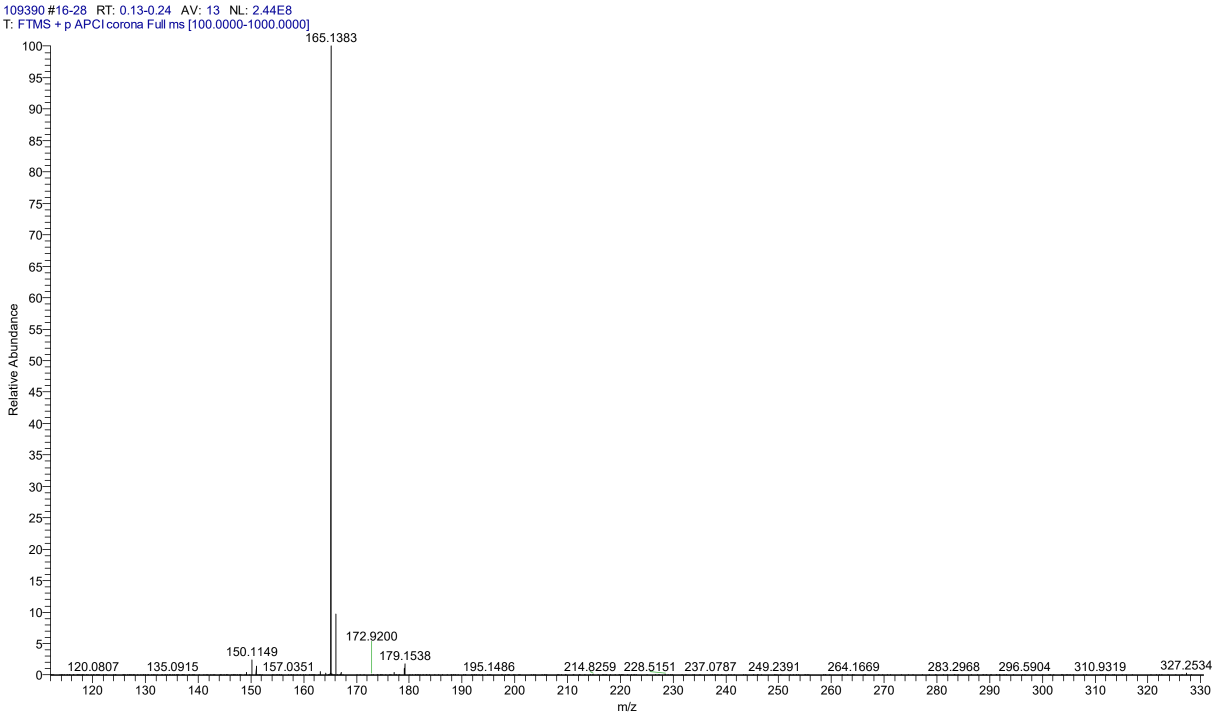

Fig. S27 GC-MS spectrum of TMB-1,3 obtained by electrospray APCI. Target m/z = 164.13.

N1,N1,N4,N4-tetramethylbenzene-1,4-diamine (TMB-1,4)

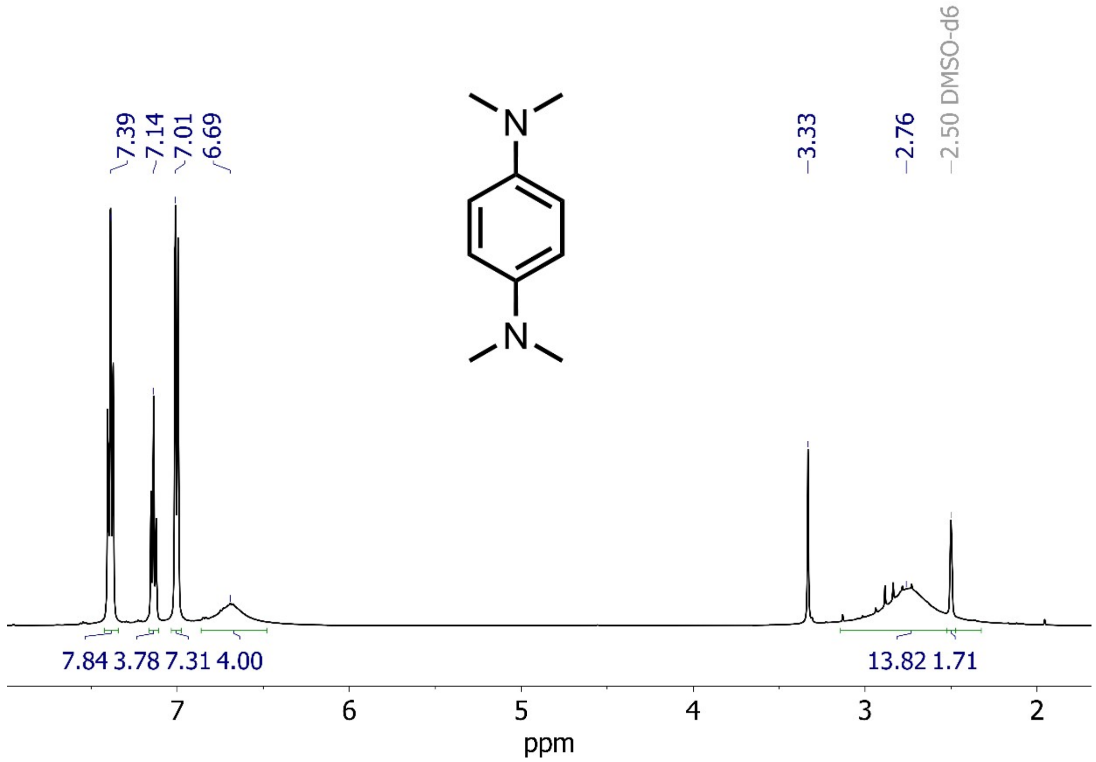

Fig. S28 ^{1}H-NMR spectrum of catalyst TMB-1,4. (500 MHz, DMSO) δ 6.69 (s, broad, 4H), 2.76 (s, broad, 12H). Note: the peaks at 7.01, 7.14 and 7.39 ppm correspond to diphenyl ether, used as an internal calibrant during quantification.

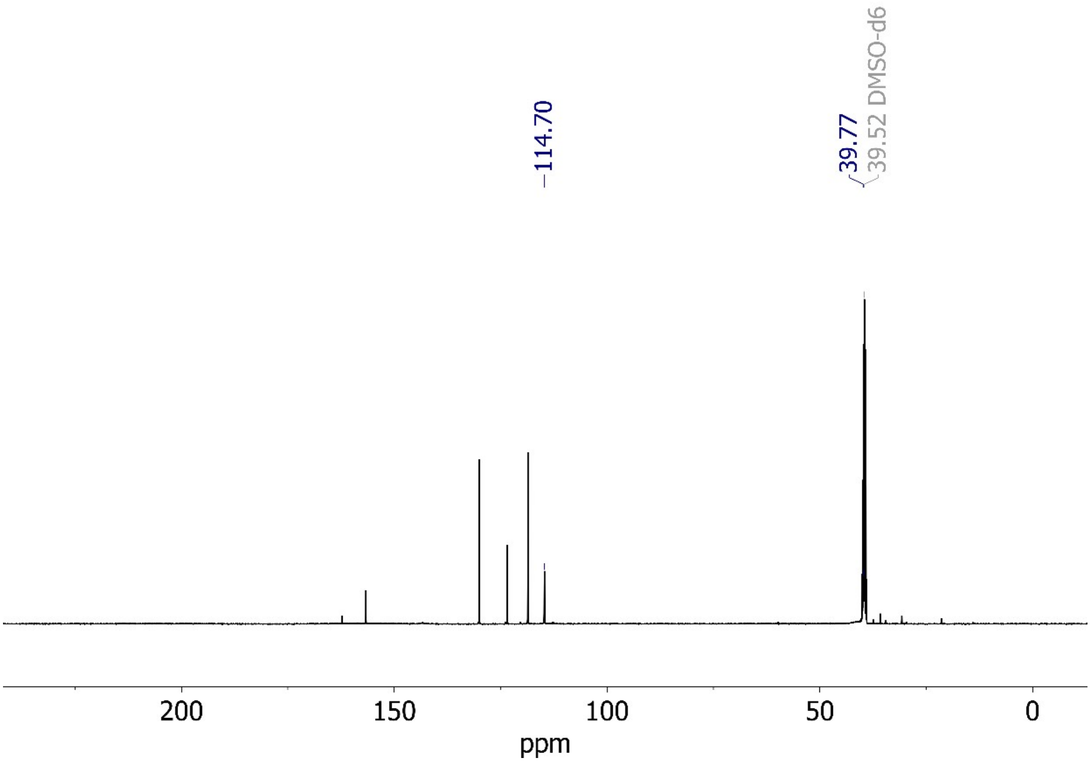

Fig. S29 ^{13}C-NMR spectrum of catalyst TMB-1,4. (126 MHz, DMSO) δ 114.70, 39.77.

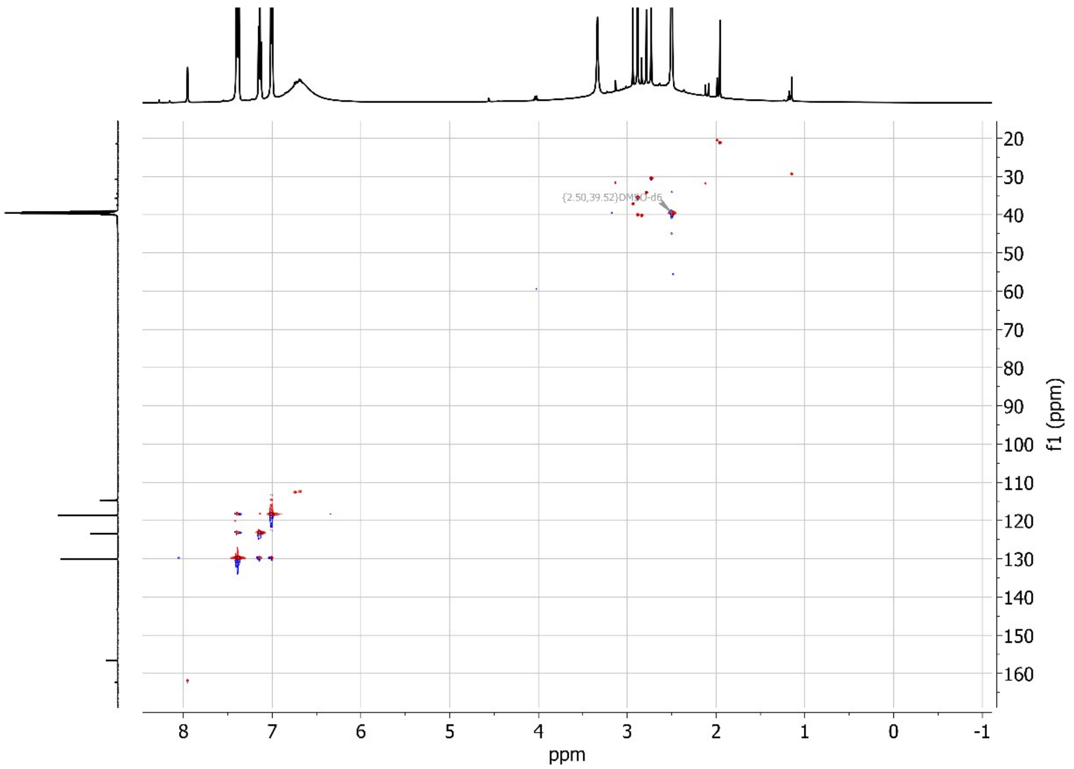

Fig. S30 HSQC NMR spectrum of catalyst TMB-1,4.

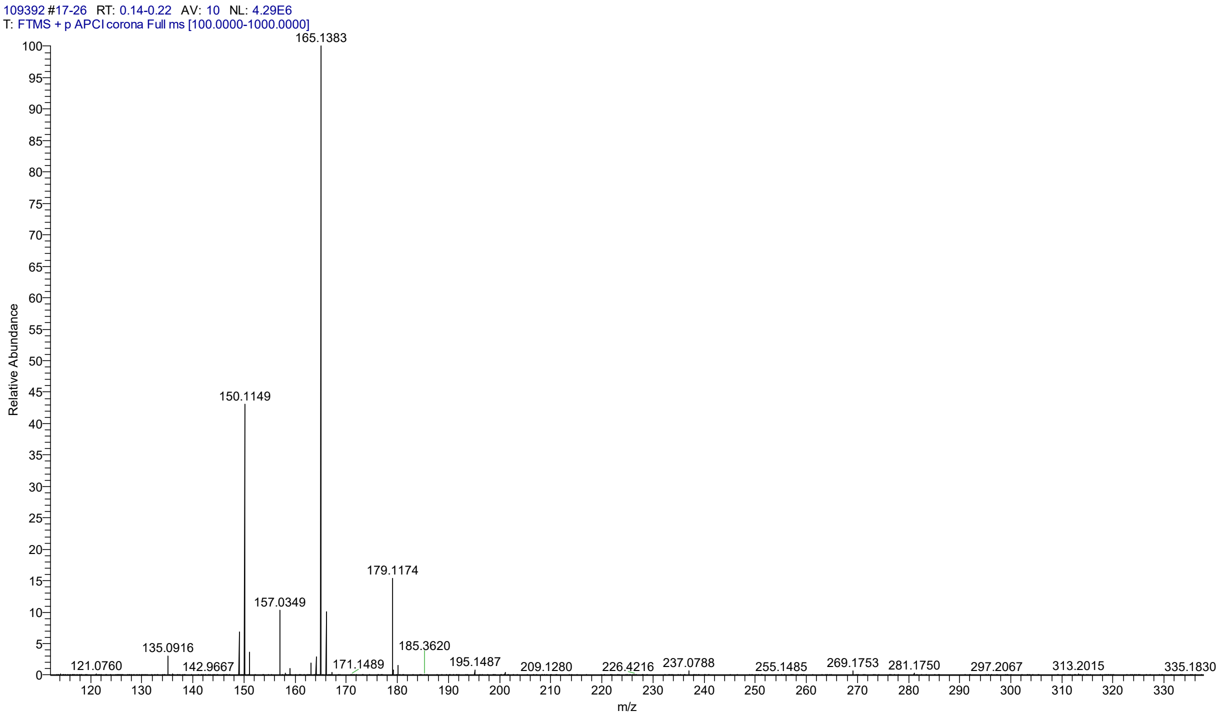

Fig. S31 GC-MS spectrum of TMB-1,4 obtained by electrospray APCI. Target m/z = 164.13.

Table S3 Comparison of catalyst library compound's pK_as and their glycolysis activity.

Catalyst	pK_a (3)	BHET yield at 4 h (%)
TMN-1,5	4.1*	5
DMQ-8	n/a	29
TMB-1,3	5.4*	10
TMB-1,4	6.2*	40
TMB-1,2	7.4*	21
3-PMB-1,2	n/a	57
Proton Sponge	12.0	89

*pK_a is in 80:20 H_2O:EtOH at 20 °C. All yields measured at 20 mol% catalyst, 10 eq. EG, 0.3 g PET, 180 °C, N_2. For PS pK_a is in water.

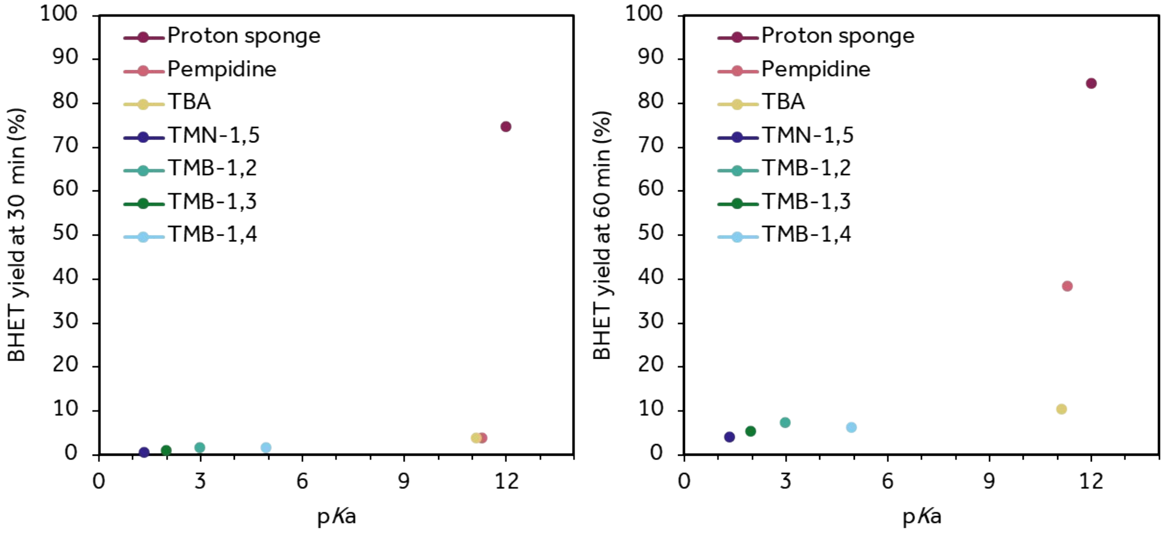

Fig. S32 Correlation between basicity and activity for the catalyst library, Proton Sponge, pempidine and tributylamine. For PS, pempidine and TBA, the pK_a values are in H_2O whereas for the library are in 20% EtOH/H_2O.

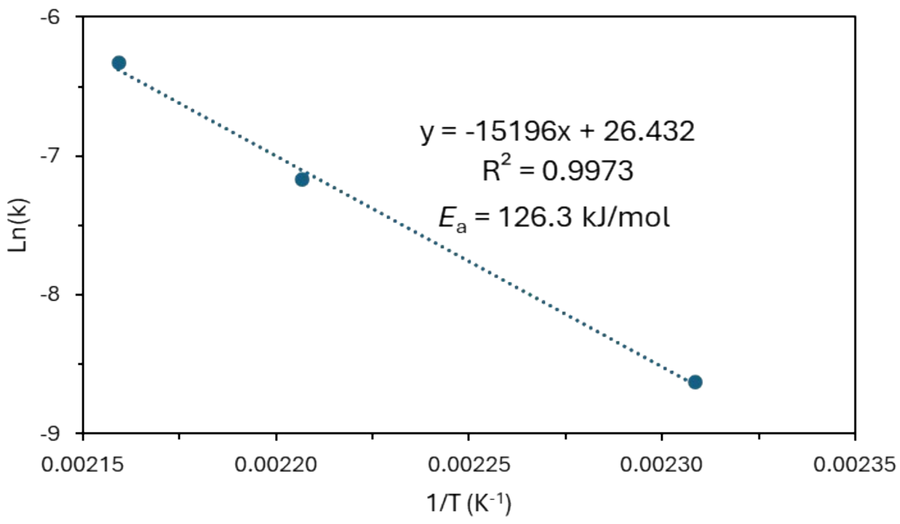

Fig. S33 Arrhenius plot of Proton Sponge-catalysed glycolysis with apparent activation energy (160, 180 and 190 °C, 10 equiv. EG, 10 mol% catalyst, N_2, 0.3 g PET)

References

(1) Sutton, J.; Grause, G.; Hmayed, A. A. R.; Street, S. T.; Dove, A. P.; Wood, J. Organocatalytic glycolysis of polyethylene terephthalate and product separation by membrane filtration. *Chem. Eng. J.* **2025**, *512*, 162400.

(2) Boutevin, B.; Ameduri, B.; Elidrissi, A.; Touzani, R. Synthesis and properties of long-chain aromatic telechelic monodispersed diols radical-initiated, addition of 2-mercaptoethanol onto α, ω nonconjugated dienes. *Phosphorus, Sulfur, and Silicon and the Related Elements* **2012**, *187* (4), 482-494.

(3) Vlasenko, M. P.; Ozeryanskii, V. A. One-scale basicities of diaminobenzenes and diaminonaphthalenes: from aniline to proton sponge. *J. Phys. Org. Chem.* **2017**, *30* (2), e3609.
